# Canine Mucosal Artificial Colon: development of a new colonic in vitro model adapted to dog sizes

**DOI:** 10.1007/s00253-023-12987-2

**Published:** 2024-01-23

**Authors:** Charlotte Deschamps, Sylvain Denis, Delphine Humbert, Nathalie Priymenko, Sandrine Chalancon, Jana De Bodt, Tom Van de Wiele, Ignacio Ipharraguerre, Inma Alvarez-Acero, Caroline Achard, Emmanuelle Apper, Stéphanie Blanquet-Diot

**Affiliations:** 1 UMR 454 MEDIS, Université Clermont Auvergne, INRAE, Clermont-Ferrand, France; 2 Lallemand Animal Nutrition, Blagnac, France; 3 Dômes Pharma, Pont-du-Château, France; 4 Toxalim (Research Center in Food Toxicology), University of Toulouse, INRAE, ENVT, INP-Purpan, UPS, 31000 Toulouse, France; 5 Center for Microbial Ecology and Technology (CMET), Faculty of Bioscience Engineering, Ghent University, Ghent, Belgium; 6 Institute of Human Nutrition and Food Science, University of Kiel, Kiel, Germany; 7 Institute of Food Science, Technology and Nutrition, Spanish National Research Council, ICTAN-CSIC), Madrid, Spain

**Keywords:** Large intestine, In vitro gut model, Body weight, Microbiota, Mucus, Breed

## Abstract

**Abstract:**

Differences in dog breed sizes are an important determinant of variations in digestive physiology, mainly related to the large intestine. In vitro gut models are increasingly used as alternatives to animal experiments for technical, cost, societal, and regulatory reasons. Up to now, only one in vitro model of the canine colon incorporates the dynamics of different canine gut regions, yet no adaptations exist to reproduce size-related digestive parameters. To address this limitation, we developed a new model of the canine colon, the CANIne Mucosal ARtificial COLon (CANIM-ARCOL), simulating main physiochemical (pH, transit time, anaerobiosis), nutritional (ileal effluent composition), and microbial (lumen and mucus-associated microbiota) parameters of this ecosystem and adapted to three dog sizes (i.e., small under 10 kg, medium 10–30 kg, and large over 30 kg). To validate the new model regarding microbiota composition and activities, in vitro fermentations were performed in bioreactors inoculated with stools from 13 dogs (4 small, 5 medium, and 4 large). After a stabilization period, microbiota profiles clearly clustered depending on dog size. *Bacteroidota* and *Firmicutes* abundances were positively correlated with dog size both in vitro and in vivo, while opposite trends were observed for *Actinobacteria* and *Proteobacteria*. As observed in vivo, microbial activity also increased with dog size in vitro, as evidenced from gas production, short-chain fatty acids, ammonia, and bile acid dehydroxylation. In line with the 3R regulation, CANIM-ARCOL could be a relevant platform to assess bilateral interactions between food and pharma compounds and gut microbiota, capturing inter-individual or breed variabilities.

**Key points:**

• *CANIM-ARCOL integrates main canine physicochemical and microbial colonic parameters*

• *Gut microbiota associated to different dog sizes is accurately maintained in vitro*

• *The model can help to move toward personalized approach considering dog body weight*

**Supplementary Information:**

The online version contains supplementary material available at 10.1007/s00253-023-12987-2.

## Introduction

Digestion is a complex and regionalized process involving physicochemical, mechanical, and microbial mechanisms and, as for other mammals, is acknowledged as a crucial element in canine health, with an increased awareness of the central role of gut microbiota (Redfern et al. 2017; Mondo et al. 2019). Since dogs are facultative carnivores, their digestion is processed by a short and simple gastrointestinal tract adapted to high-protein and high-fat diets (Kararli 1995). More than 400 canine breeds have been genetically selected by humans, leading not only to huge variations in size, weight, and appearance, but also to changes in digestive anatomy and genetic profile adaptation, such as the apparition of amylase-encoding gene (Axelsson et al. 2013; Botigué et al. 2017). How canine digestion is influenced by dog size was recently reviewed (Deschamps et al. 2022a), highlighting that most of the identified variations were related to the large intestinal compartment. Large intestine length, area, and volume were shown to increase with dog weight, in association with a higher colonic transit time. In close relation with this longer transit time, fermentative capacity and especially fiber degradation seem to be amplified with body weight (Weber et al. 2002b; Detweiler et al. 2019; Nogueira et al. 2019), resulting in an important production of short-chain fatty acids (SCFAs) in large dogs and a lower colonic pH (Weber et al. 2004). Fecal concentrations of phenol, indole, ammonium, and branched-chain fatty acids (BCFAs) were also positively correlated with body weight, again probably in relation to the longer colonic transit time favoring protein fermentation (Goudez et al. 2011; Beloshapka et al. 2012, 2014; Alexander et al. 2019). This is in accordance with an increased abundance of fecal *Fusobacteria* with body weight (Gazzola et al. 2017; Kim et al. 2017; Algya et al. 2018). Fecal bile acid profiles are also impacted by dog sizes, with an apparent decrease of total bile acids, as well as primary to secondary bile acid ratios, when body weight increases (Guard et al. 2019). Lastly, the intestinal mucosa of large dogs is also characterized by a higher permeability which could induce a backflow of absorbed electrolytes and explain their poorer fecal consistency (Bjarnason et al. 1995; Zentek and Meyer 1995; Weber et al. 2002a, 2017). In-depth characterization of the variations with dog sizes of colonic physicochemical parameters and microbiota composition and functions is of high interest, especially because those factors can reshape not only essential processes such as nutrient digestibility but also drug bio-accessibility (notably for colon-targeted formulations) or probiotic/enteric pathogen survival and activity.

Even if in vivo experiments still remain the ultimate goal in nutrition or pharma studies, the use of animals in research is more and more limited by ethical, regulatory, and cost reasons. The number of dogs used in research and testing has a decrease of 26 % between 2018 and 2019, but 13,076 dogs were still involved in European countries in 2019 (European Commission 2022). Among them, more than 2000 were used for legislation on medicinal products for veterinary use and over 8100 for human use (European Commission 2022). The European 3Rs rules widely encourage the use of alternative strategies such as in vitro models reproducing digestion or fermentation processes occurring within the gut. Up to now, most of the in vitro systems developed to reproduce the canine large intestine are simple static batch models (Sunvold et al. 1995; Tzortzis et al. 2004; Bosch et al. 2008; Cutrignelli et al. 2009; Panasevich et al. 2013; Vierbaum et al. 2019; Van den Abbeele et al. 2020; Oba et al. 2020). These models are inoculated with dog stools, but most of them have not been adapted to the canine specific digestive environment. In addition, they cannot simulate colonic transit time and are limited to 24–72 h experiments due to a shift in major parameters such as pH, preventing the evaluation of repeated administration of drugs or food compounds. Only one dynamic model called M-SCIME (Mucosal Simulator of the Canine Intestinal Microbial Ecosystem) was very recently developed to reproduce the canine GI tract from the stomach to the large intestine, with a distinction between the luminal and mucus-associated microbiota in the colon (Duysburgh et al. 2020; Verstrepen et al. 2021). This model is based on semi-continuous fermentation processes in the three colonic parts, allowing to reproduce in vivo parameters such as pH evolution, transit time, supply of non-digested nutrients, and anaerobiosis by continuous N_2_ flushing. However, up to date, none of the available models, including the M-SCIME, was set up to differentiate colonic conditions depending on dog’s size.

In this context, the aim of this study was to develop and in vivo validate a new in vitro model of the canine colon adapted to dog body weight, as a relevant alternative to animal assays. Based on the continuous fermentation Mucosal Artificial Colon Model (M-ARCOL) (Deschamps et al. 2020), the newly developed system aims to accurately reproduce both the physicochemical, nutritive, and microbial parameters specific to the colonic ecosystem of three different dog’s sizes, namely “small” (under 10 kg), “medium” (from 10 to 30 kg), and “large” (over 30 kg).

## Materials and methods

### Fecal sample collection and treatment

**Fig. 1:**
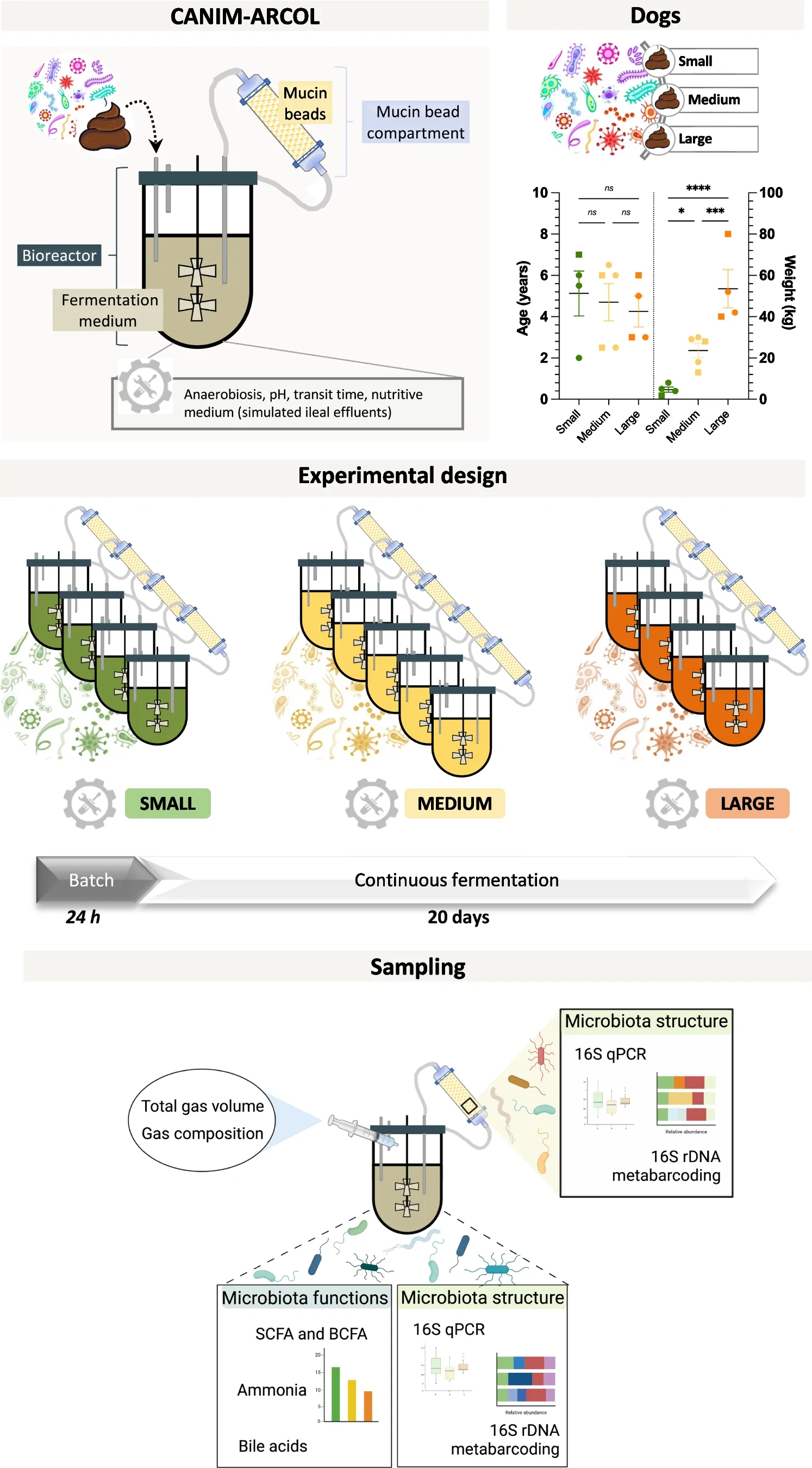
Overview of experimental design in the CANIM-ARCOL model. Once adapted to three dog sizes conditions, the CANIM-ARCOL was inoculated with fecal samples from 13 healthy dogs (*n* = 4 small in green, *n* = 5 medium in yellow and *n* = 6 large in orange) and fermentations were run for 21 days. Age and weight of all dogs involved in the study (males and females are represented by square and circle, respectively) were plotted, and significant differences were analyzed by one-way ANOVA (mean ± SD, single asterisk: *p* < 0.05; triple asterisk: *p* < 0.001; quadruple asterisks: *p* < 0.0001). Samples were regularly collected in the atmospheric phase, in the luminal compartment and from mucin beads to monitor microbiota composition and metabolic activities.

Feces from 13 clinically healthy dogs of several breeds, ages, and weights (see Supplemental Table S1 and Fig. 1 for details) were collected. All dogs were adult owner-pets and fed with commercial dry food. They had a body score condition of 3 under a 5-point scale, meaning all of them had a normal body weight. Immediately after defecation, fecal samples were transferred into a sterile recipient, placed in an airtight anaerobic box (GENbag anaer gas pack systems, Biomerieux, France), transported, and frozen at −80 °C within 6 h until processing in the next 24 h. In an anaerobic chamber (COY laboratories, Grass Lake, MI, USA), stool samples were manually homogenized, and 3.3 g of feces was resuspended with 30 mM sterile sodium phosphate buffer (pH 6.0) to reach a total volume of 100 mL. Because of the small amount of feces provided by small size dogs, only 1.8 g can be resuspended into 100 mL for donor S1 and 2.1 g for donors S2, S3, and S4. Feces were then mixed and filtered using a 500-µm inox sieve.

### Description and parameters of CANIM-ARCOL model

The newly developed Canine Mucosal Artificial Colon (CANIM-ARCOL) was adapted from the one stage continuous fermentation system previously set-up under human condition and called M-ARCOL (Deschamps et al. 2020). The in vitro model allows to reproduce both luminal and mucosal phases of the large intestine by the use of respectively a bioreactor (MiniBio, Applikon, Delft, The Netherlands) and an airtight glass vessel connected to the bioreactor and containing mucin beads (Fig. 1). At the beginning of the experiment, 100 mL of fecal suspension was added per bioreactor to 200 mL of canine-adapted nutritive medium simulating the composition of ileal effluents (Table 1) and containing various sources of carbohydrates, proteins, lipids, minerals, and vitamins. After an initial sparging of O_2_-free N_2_-gas, anaerobiosis was maintained during the total course of the fermentation by the sole activity of the resident microbiota and through ensuring the system airtightness. Bioreactor was kept at body temperature. Colonic pH and redox potential were constantly recorded (Applikon, Delft, The Netherlands), and pH was adjusted with 2 M NaOH. The nutritive medium was continuously introduced into the bioreactor, while the fermentation medium was automatically withdrawn, ensuring the appropriate mean retention time and maintaining the colonic volume constant. The mucosal compartment was dived in a water bath maintained at body temperature. It was filled with mucin-alginate beads offering an overall surface of 556 cm^2^ in average. To produce mucin beads, mucin type II from porcine stomach (Sigma-Aldrich, Saint-Louis, MO, USA) and sodium alginate (Sigma-Aldrich, Saint-Louis, MO, USA) were diluted in sterile distilled water, at concentrations of 5 % and 2 % respectively and pH was adjusted to 6 with a sodium bicarbonate solution. The mucin/alginate solution was dropped using a peristaltic pump into a sterile solution of 0.2 M CaCl_2_ dihydrate under agitation. Every 2 days, mucin/alginate beads remaining into the mucosal compartment were renewed by fresh sterile ones under a constant flow of CO_2_ to avoid oxygen entrance.

**Table 1:**
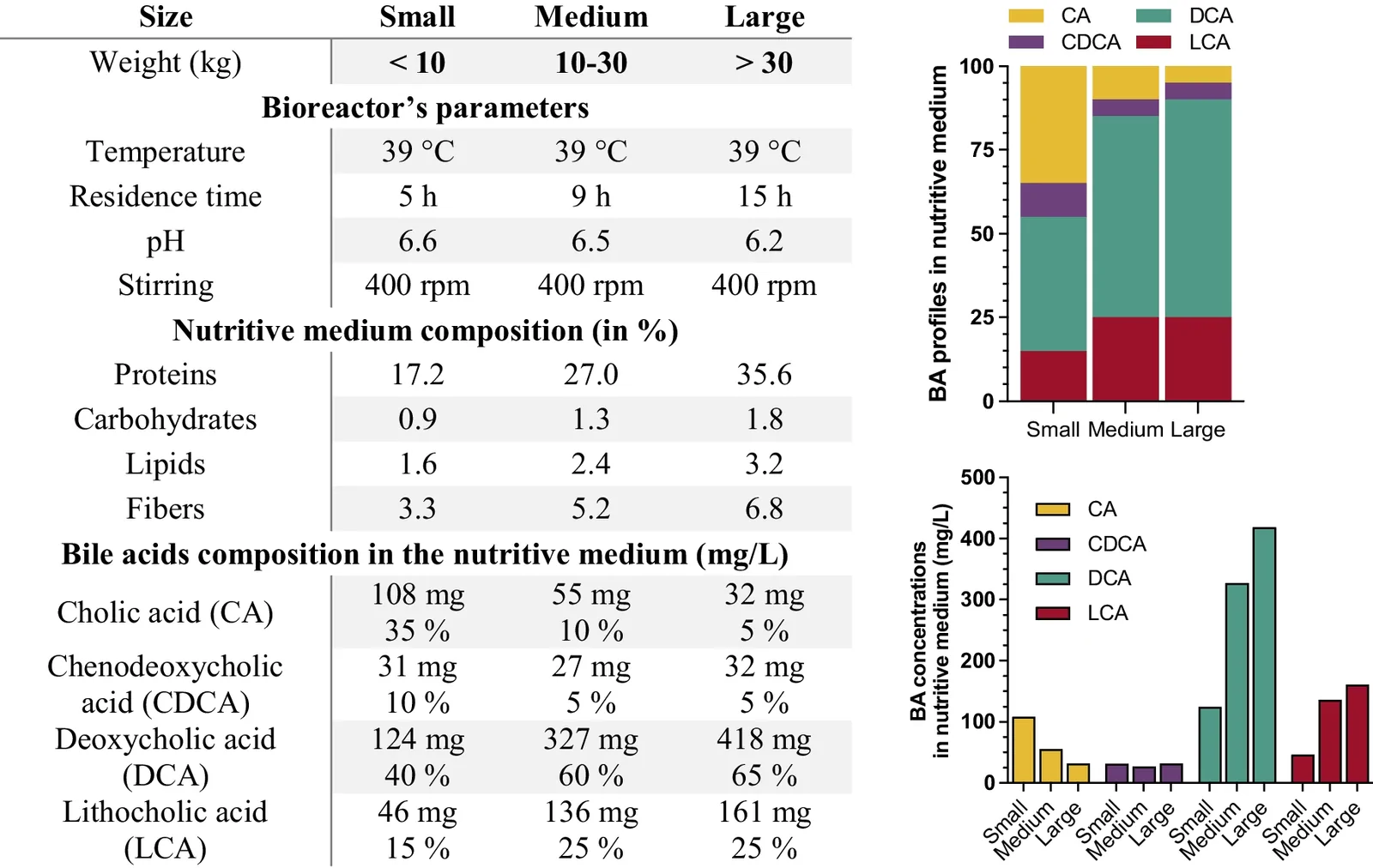
Adaptation of physicochemical and nutritional parameters of CANIM-ARCOL to three dog sizes

In the present study, the in vitro colon model was adapted to reproduce the mean conditions found in the large intestine of healthy adult dogs and adapted to three canine sizes as summarized in Table 1 and detailed in the result part. Briefly, temperature was set at 39 °C. Residence time and pH were revised according to in vivo data and set up at 5 h and pH 6.5 for small, 9 h and pH 6.5 for medium, and 15 h and pH 6.2 for large dog conditions. The composition of the nutritive medium was also adapted to three dog size diet and digestive physiology.

### Experimental design and sampling

Experimental design in the CANIM-ARCOL is presented in Fig. 1. In all the experiments, each bioreactor was inoculated with a fecal sample from a unique donor (from either small, medium, or large dogs). Bioreactors corresponding to one dog size were run in parallel (*n* = 4 for small and large conditions and *n* = 5 for medium ones) and set up with specific parameters. Fermentations were run under batch conditions for 24 h and then under continuous conditions for 20 additional days. Samples were collected daily in the fermentative medium (corresponding to the luminal phase) for analysis of microbiota composition (qPCR and 16S rRNA Metabarcoding) and metabolic activities through short-chain fatty acids (SCFA), branched chain fatty acids (BCFA), total bile acids, and ammonia dosage. The atmospheric phase was also sampled to follow anaerobiosis and evaluate gas composition and production thanks to a sampling bag connected to the condenser (Fig. 1). Every 2 days, mucin beads were collected for mucus-associated microbiota analysis (qPCR and 16S rRNA Metabarcoding). Mucin beads were washed twice in sterile sodium phosphate buffer and stored at −80 °C before downstream analysis.

### DNA extraction

Genomic DNA was extracted from fermentative medium samples and mucin/alginate beads using the QIAamp Fast DNA Stool Mini Kit (Qiagen, Hilden, Germany) following manufacturer’s instructions with the following modifications (Deschamps et al. 2020). Prior to DNA extraction, luminal samples were centrifuged (18,000 rcf, 15 min, 4 °C) and the pellets were collected. Pellets and mucin/alginate bead samples were then incubated for 10 min with sterile citrate buffer (sodium citrate 55 mM and NaCl 154 mM) at 37 °C (Capone et al. 2013), before vortexing (maximal speed, 15 s), followed by centrifugation again (8000 rcf, 1 min). Then, a step of mechanical disruption using a bead beater (5 min, 20 beat/s) was made with 300-mg sterile glass beads (diameter ranging from 100 to 600 µm). DNA quantity was evaluated using the Qubit dsDNA Broad Range Assay Kit (Invitrogen, Carlsbad, CA, USA) with a Qubit 3.0 Fluorometer (Invitrogen, Carlsbad, CA, USA). Samples were stored at −20 °C prior to microbiota analysis (qPCR and 16S rRNA Metabarcoding).

### Quantitative PCR

Total bacteria were quantified by qPCR using primers described in Supplemental Table S2. Real-time PCR assays were performed in a Biorad CFX96TM Real-Time System (Bio-Rad Laboratories, Hercules, CA, USA) using Takyon Low ROX SYBR 2X MasterMix blue dTTP kit (Eurogentec, Seraing, Belgium). Each reaction was run in duplicate in a final volume of 10 μL with 5 μL of MasterMix, 0.45 μL of each primer (10 μM), 1 μL of DNA sample, and 3.1 μL of ultra-pure water. Amplifications were carried out using the following ramping profile: 1 cycle at 95 °C for 5 min, followed by 40 cycles of 95 °C for 30 s and then 58 °C for 30 s. A melting step was added to ensure primer specificity. Standard curve was generated from 10-fold dilutions of bacterial DNA (isolated from a pure culture of bacteria), allowing the calculation of DNA concentrations from extracted samples.

### 16S rRNA metabarcoding and data analysis

Bacterial V3-V4 regions of 16S ribosomal RNA (rRNA) and the archaeal 16S rRNA were amplified using primers described in Supplemental Table S2. Amplicons were generated using a Fluidigm Access Array followed by high throughput sequencing on an Illumina MiSeq system (Illumina, IL, USA) performed at the Carver Biotechnology Center of the University of Illinois (Urbana, USA). Bioinformatic analysis was performed using R studio software and helped by rANOMALY package (Theil and Rifa 2021). Prior to analysis, raw data were demultiplexed and quality filtered using DADA2 R-package (Callahan et al. 2016). Reads with quality score under 2 were truncated. Reads under 100 bp length were removed as well as sequences similar to PhiX DNA used as a spike-in control for MiSeq runs. Filtered sequences were dereplicated and cleaned for chimeras (DADA2). Taxonomic classification of the sequences was then performed with DECIPHER package (Murali et al. 2018). Assignations from both SILVA 138 release (Quast et al. 2013) and GTDB_bac120_arc122 (Parks et al. 2022) databases were merged using the assign_taxo_fun function from rANOMALY R-package, based on IDTAXAusing IDTAXA with a 60% confidence cutoff. A phylogenetic tree was then reconstructed using DECIPHER.

### Gas analysis

The analysis of O_2_, N_2_, CO_2_, CH_4_, and H_2_ gas produced during the fermentation process was performed using 490 micro-gas chromatography (Agilent Technologies, Santa Clara, CA, USA) coupled with a micro-TCD detector (Agilent Technologies, Santa Clara, CA, USA). Molecular Sieve 5A and Porapack Q (Aligent Technologies, Santa Clara, CA, USA) series columns were used. Gas composition was determined using calibration curves made from ambient air (78.09 % N_2_, 20.95 % O_2_, 0.04 % CO_2_) and three gas mixtures A (5% CO_2_, 5 % H_2_, 90 % N_2_), B (20 % CO_2_, 80 % H_2_), and C (20 % CO_2_, 20 % CH_4_, 20 % H_2_, 40 % N_2_). Technical replicates were performed for each sample, and results were expressed as relative percentages.

### Fatty acid analysis

For SCFA analysis, 1.5 mL of luminal samples was centrifuged (18,000 rcf, 15 min, 4 °C) and 900 μL of supernatant was diluted at 1/10 into H_2_SO_4_ 0.04 M mobile phase, vortexed, and filtered (0.22 μm). The three major SCFAs (acetate, propionate, and butyrate) were quantified by high-performance liquid chromatography (HPLC) (Elite LaChrom, Merck HITACHI, Westford, MA, USA) coupled with a DAD diode. The HPLC column (Concise Separations, San Jose, CA, USA, ICE-99-9865) and its guard column were maintained at 50 °C. Sulfuric acid 0.04 M was used as mobile phase, and SCFAs were separated at a flow rate of 0.6 mL/min. Data were obtained and analyzed by the EZChrom Elite software (Agilent, Santa Clara, CA, USA) at 205 nm. SCFA concentrations were calculated from calibration curves established from known concentration solutions of acetate, propionate, and butyrate (0, 10, 25, and 40 mM), and data were expressed as millimolar or relative percentages. BCFAs (isobutyrate, isovalerate, valerate, isocaproate, caproate, heptanoate) were measured by gas chromatography (GC-2014, Shimadzu®, Hertogenbosch, The Netherlands) with a DB-FFAP 123-3232 column (30 m × 0.32 mm × 0.25 µm; Agilent, Santa Clara, CA, USA) and a flame ionization detector (FID). Liquid samples were conditioned with sulfuric acid and sodium chloride and 2-methyl hexanoic acid as internal standard for quantification and further extracted with diethyl ether. Prepared sample (1 µL) was injected at 280 °C with a split ratio of 60 and a purge flow of 3 mL/min. The oven temperature increased by 6 °C/min from 110 to 158 °C and by 8 °C/min from 158 to 175 °C where it was kept for 1 min. FID had a temperature of 220 °C. The carrier gas was nitrogen at a flow rate of 2.49 mL/min. BCFA concentrations were calculated from calibration curves established using known concentrations of pure solutions of each fatty acid, and the data were expressed as millimolar or relative percentages.

### Bile acids quantification and ammonia dosage

For bile acid extraction, water:acetonitrile (1:1) and CDCA-d4 as the internal standard were used. Bile acids were quantified by HPLC-QQQ-MS, employing as external standards cholic acid (CA), deoxycholic acid (DCA), lithocholic acid (LCA), chenodeoxycholic acid (CDCA), glycodeoxycholic acid (GDCA), glycolic acid (GCA), taurodeoxycholic acid (TDCA), taurocholic acid (TCA), hyodeoxycholic acid (HDCA), ursodeoxycholic acid (UDCA), and hyocholic acid (HCA) in a range of concentrations between 5 and 0.001 µg/mL. The separation was done using a Phenomenex Kinetex XB-C18 100A column, with ammonium acetate 2 mM in water and acetonitrile:methanol (1:1) as mobile phases and a constant flow rate of 1 mL/min at a temperature of 50 °C. The HPLC used was an Agilent 1200 coupled to a Triple Quadrupole (QQQ) Agilent (Agilent, Santa Clara, CA, USA, G6410B). Data processing was performed with Masshunter Qualitative Analysis (Agilent, Santa Clara, CA, USA, version B.07.00), and quantification was performed in the multiple reaction monitoring (MRM) mode by integration of ion areas based on standard curves using authentic standards and chenodeoxycholic acid-d4 as internal standard (IS).

Total ammonia was measured using the total bile acid enzymatic test (Diazyme Laboratories, Poway, USA) following manufacturer’s instructions. Results were expressed in millimolar.

### Statistical analysis

Statistical analyses on microbiota activity (gas, SCFA, BCFA, ammonia, total bile salts) and α-diversity indexes (number of observed amplicon sequence variants (ASVs) and Shannon index) from 16S rRNA Metabarcoding data were processed using GraphPad Prism software version 9.4.1 (GraphPad Software, San Diego, CA, USA). Data normal distribution was verified by combining Anderson-Darling, D’Agostino & Pearson, Shapiro-Wilk, and Kolmogorov-Smirnov tests, and homoscedasticity was checked using the Fisher test. Then, appropriate statistical analysis was applied (either one-way ANOVA, *t*-test, Mann-Whitney, or Welch’s tests). First, principal coordinate analysis (PCoA, data not shown) was performed followed by non-metric multidimensional scaling (NMDS), highlighting important size and microenvironment (i.e., luminal medium and mucin beads) effects. Constraint redundancy analysis (RDA) was then performed with age, weight, sex, size, microenvironment, donor, and time as variables if the model. The analysis was conducted first with all parameters and then with the removal of either size or microenvironment variables. Bray-Curtis distances were used for each analysis, and significance between groups was assessed with a one- or two-way ANOVA. Discriminant analyses (sparse partial least squares discriminant analysis, sPLS-DA) were finally performed using MixOmics package (Lê Cao et al. 2009).

## Results

### Characterization of canine donors and fecal microbiota

Stool samples from 13 adult dogs (including 5 females and 8 males), gathered into three size groups as previously defined (i.e., small, medium, and large), were collected to inoculate CANIM-ARCOL bioreactors. Mean body weights of the dogs were respectively 4.7 ± 1.3, 23.6 ± 3.4, and 53.5 ± 9.2 kg for small, medium, and large dogs with, as expected, significant differences between those three groups (Fig. 1). Mean ages were respectively 5.1 ± 1.0, 4.7 ± 0.9, and 4.3 ± 0.8 years, with no significant differences between groups (Fig. 1).

Microbiota composition and key microbial metabolites were characterized in the initial stools as a global description of the biological material used for bioreactor inoculation (Supplemental Fig. S1). 16S rRNA metabarcoding analysis showed at phylum level a higher abundance of *Firmicutes* and a lower amount of *Fusobacteriota* in the large dogs compared to other sizes (Supplemental Fig. S1a). A high variability in microbial profiles at the family level was also noticed between different dog sizes and between individuals (Supplemental Fig. S1d), body weight being the predominant explanatory variable (*p* = 0.001) for dissimilarities in fecal microbiota composition (Supplemental Fig. S1b). Interestingly, total fecal SCFA increased with dog size (*p* > 0.05, Supplemental Fig. S1e), while BCFA showed opposite trends (*p* < 0.0001; Supplemental Fig. S1g), and no clear tendency was observed for ammonia (Supplemental Fig. S1i) and total bile acids (data not shown). Profiles obtained for SCFAs (Supplemental Fig. S1f), BCFAs (Supplemental Fig. S1h), and bile salts (Supplemental Fig. S1j) also showed size-dependent effects. Note that heptanoic acid was found only in the fecal samples from small dogs (Supplemental Fig. S1h).

### Setup of the in vitro model with specific canine size–related colonic parameters

#### Colonic physicochemical parameters

Temperature was set at 39 °C for all size groups according to veterinary recommendations. Regarding pH, there is no study evaluating colonic pH of small and large dogs and only two in medium dogs. Koziolek et al. (2019) described a pH from 5 to 8, and Smith (1965) reported a pH of 6.5, but none specified dog diet even if it is well known to influence gastrointestinal pH (Scarsella et al. 2020). From our previous literature review (Deschamps et al. 2022a), we determined fecal pH mean values of 6.6, 6.5, and 6.2 respectively for small, medium, and large dogs. Since these data are in accordance with the negative correlation between fecal pH and canine body weight described in the literature (reviewed in Deschamps et al. (2022a)), and the value for medium dogs was in line with that of Smith (1965), those three values were chosen for the model set-up (Table 1). Lastly, a previous study showed that large intestinal transit time can be estimated as a percentage of total transit time, with a positive correlation between large intestinal transit time and body weight (Hernot et al. 2006). Authors established that the large intestinal transit time represents 40, 55, and 70% of total transit time for small, medium, and large dogs, respectively. Applied to the mean total transit times established from our literature review for each dog size (in total 23 studies (Deschamps et al. 2022b), we calculated average large intestinal transit times of 10, 18, and 30 h for small, medium, and large dogs, respectively. Those estimations were fully in line with in vivo data from studies which estimated this digestive parameter in various dog sizes (Bruce et al. 1999; Hernot et al. 2006; Boillat et al. 2010; Lidbury et al. 2012; Gazzola et al. 2017; Koziolek et al. 2019). When applied to the in vitro model, this led to residence time (time for renewal of half of fermentation medium) of 5, 9, and 15 h, respectively (Table 1).

#### Nutrient supply in simulated ileal effluents

The canine nutritive medium simulating ileal effluent composition was adapted from that initially developed for human by Macfarlane et al. (1998) and typically used in the M-ARCOL model (Deschamps et al. 2020), as summarized in Table 1. The composition was adapted to consider canine dry diet composition and energetical requirements for the three dog sizes, as well as ileal digestibility indices for each type of nutrients. Calculations were based on a mean energy requirement of respectively 730, 1160, and 1523 kcal/day for small, medium and large dogs (Case et al. 2011). Quantities of nutrients supplied to the in vitro model (simulated ileal effluents) were calculated assuming that they are corresponding to the fraction that has been not digested and absorbed in the upper gut. Thus, the percentage of nutrients delivered into the colon was estimated as the difference between food intake and ileal digestibility. Since no study had evaluated the ileal digestibility of macronutrients in small and large dogs, we used the data available from medium dogs for all size conditions (Bednar et al. 2000; Flickinger et al. 2003; Propst et al. 2003; Hendriks et al. 2013), i.e., 77, 95, and 99 % of initial intake for protein, lipid, and carbohydrate, respectively. Protein sources were adapted with 80 and 20% from animal and vegetal origins, respectively, to cover the entire set of amino acids (FEDIAF 2019). Lipids were given by addition of linoleic acid (poly-unsaturated omega-6 fatty-acid) and palmitic acid (essential saturated fatty acid for dogs) (FEDIAF 2019). Regarding carbohydrate sources, purified starch from corn, rice, and wheat (1/3 each) were added in the in vitro nutritive medium based on in-field proportion (commercial dry food). The same quantity of fiber per day and per kilogram of body weight was provided for each size group, but the ratio between soluble and insoluble fibers was changed according to dog size (Weber et al. 2017). Therefore, a soluble-to-insoluble ratio of 70/30, 50/50, and 30/70 was applied for small, medium, and large conditions, respectively. Based on 2 g fibers/100 g of dry food, soluble fibers were provided in the in vitro nutritive medium by 22 % pectin, 53 % inulin, and 25 % oligosaccharides (2/3 fructo-oligosaccharides and 1/3 mano-oligosaccharides), while insoluble fibers were given by a 50/50 ratio of arabinogalactan and cellulose.

#### Bile acid content

Fecal total bile acids were quantified in all dog sizes in only one study (Guard et al. 2019) and measured 5.1, 4.7, and 3.4 µg bile acids/mg lyophilized feces for small, medium, and large dogs, respectively. The amount of total bile acids per gram of fresh stools was then estimated as 1657.5, 1428.8, and 958.8 µg for small, medium, and large dogs, respectively, based on a fecal moisture tendency curve established by Weber’s team (Weber et al. 2004). To further estimate bile acid amount transiting in the large intestine within 24 h, we considered in the calculations the colonic transit time associated to each size group and the amount of feces produced per day, i.e., 5.6 g feces/kg body weight/24 h (Algya et al. 2018). This led to a total amount of bile acids in the nutritive medium per 24 h of 445, 432, and 257.5 for small, medium, and large dogs, respectively (Table 1). Primary-to-secondary bile acid ratio of 40/60, 15/85, and 10/90 for small, medium, and large conditions, respectively, was determined according to Guard et al. (2019). Composition in major bile acids (i.e., cholic acid, chenodeoxycholic acid, lithocholic acid, and deoxycholic acid) was also estimated using Guard quantifications.

#### In vitro stabilization of colonic microbiota

**Fig. 2:**
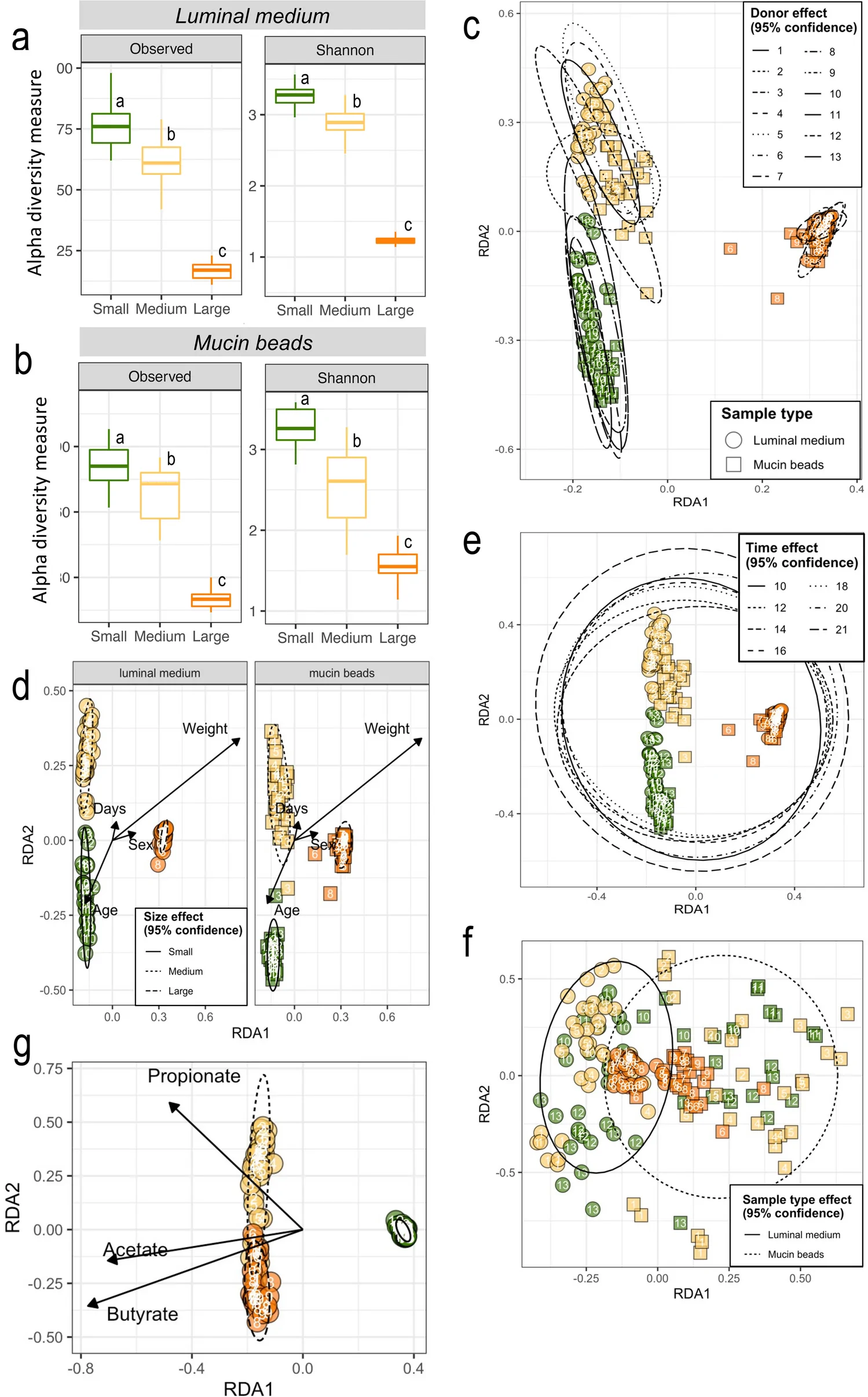
Impact of dog sizes on α and β-diversity of microbial populations in the CANIM-ARCOL model Fermentations were performed in the CANIM-ARCOL under three dog size conditions. Lumen and mucus-associated microbiota composition were analyzed by 16S rRNA Metabarcoding and diversity indexes were calculated based on ASV table. Only stabilized points (from days 10 to 21) are represented. Alpha diversity (observed ASVs and Shannon index) is represented as box plots in the luminal medium (**a**) and mucin-beads (**b**), with significant differences indicated by different letters (*p* < 0.05). Redundancy analysis (RDA) two-dimension plot visualizations reported bacterial community β-diversity, showing the effects of fermentation time (**c**), dog size (**e**), or donor effect (**f**). Size effect was removed (partial-RDA) to visualize the impact of colonic microenvironment, i.e., luminal medium or mucin beads (**d**). For luminal samples only, corresponding SCFA concentrations were added as environmental variables and RDA was recalculated accordingly (**g**). Samples from luminal medium are represented in circles while mucin beads are in squares. Numbers correspond to dog ID.

CANIM-ARCOL was used to run colonic fermentations using small, medium, or large parameters (as described above) and inoculated respectively with small, medium, and large canine stools. Gut microbiota activity and composition were followed daily to determine the time period necessary for microbiota stabilization. The stabilization state was generally reached after 7 to 10 days based on individual gas (Supplemental Fig. S2) and SCFA (Supplemental Fig. S3) profiles, even if gas profiles for most of small dogs still fluctuated at the end of fermentation (especially methane production). As expected, after stabilization, anaerobiosis was efficiently maintained by the sole activity of resident microbiota with percentages of oxygen remaining below 4 %. At the phylum level (Supplemental Fig. S4), microbiota composition was also generally stabilized after 10 days both in the luminal and mucosal compartments, even if in some donors and/or under large dog conditions, stabilization was difficult to reach. Taken together, those data indicated that at least 10 days were necessary to stabilize the canine microbiota composition and activity. Redundancy analysis (RDA) confirmed that this time period is sufficient to reach stabilization in the in vitro colon model since no significant time effect could be observed from day 10 to day 21 in both luminal and mucosal compartments, whatever the dog size (time effect was significant including days 1 to 21, data not shown) (Fig. 2c). Stabilization in the CANIM-ARCOL was also associated to a decrease in microbial richness and evenness compared to the initial stool (Fig. 2a and 2b and Supplemental Fig. S1a), as previously reported in other in vitro models (Van den Abbeele et al. 2010; Deschamps et al. 2020). The stabilization was also associated with a shift from fecal (Supplemental Fig. S1d) to colonic profiles (Supplemental Fig. S4), mainly characterized by an increase in *Bacteroidota* in accordance with in vivo data (Suchodolski et al. 2008; Honneffer et al. 2017).

### Region-specific colonic microbiota composition and inter-individual variability

**Fig. 3:**
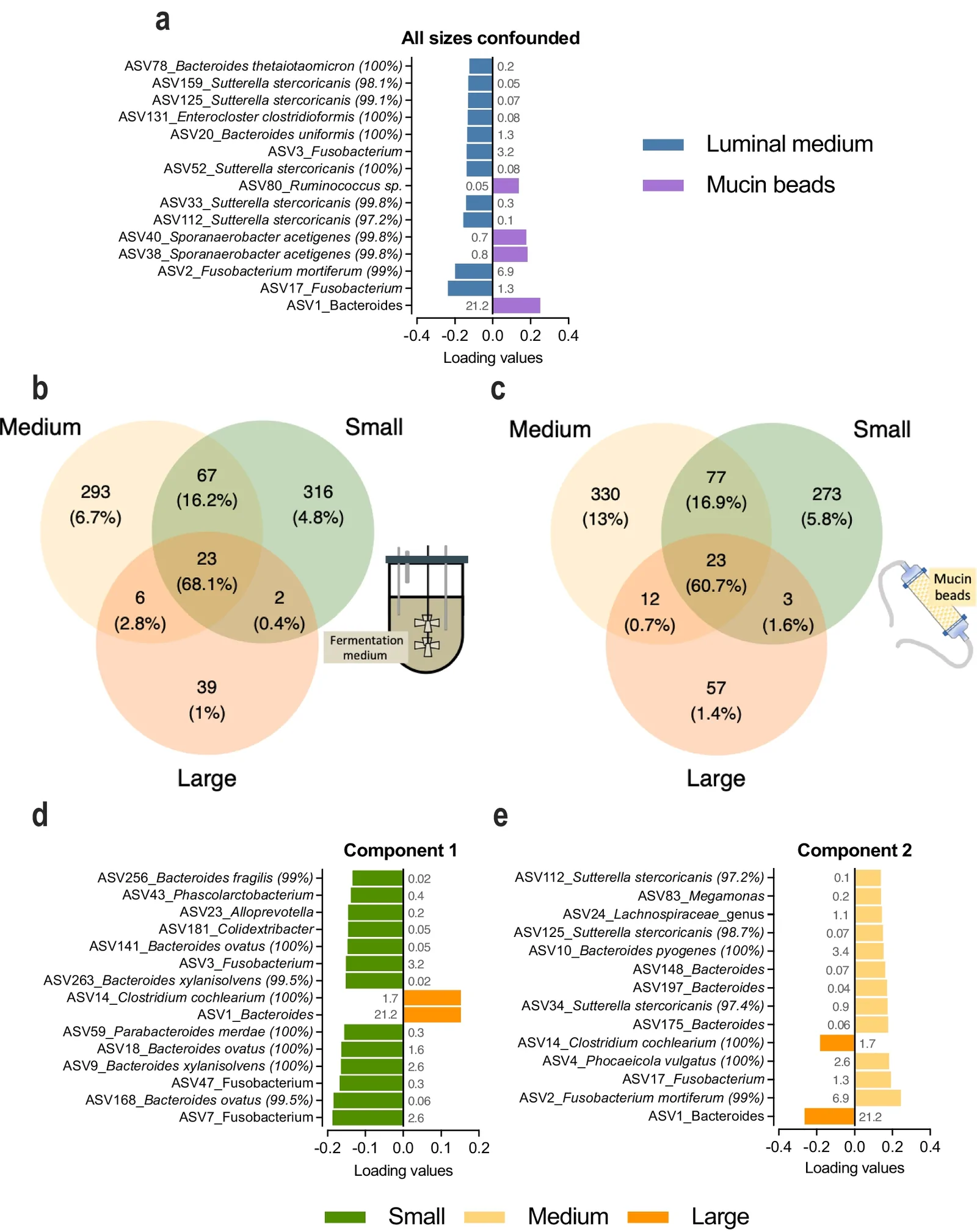
Impact of colonic microenvironment and dog size on microbiota composition in the CANIM-ARCOL. Fermentations were performed in the CANIM-ARCOL under three dog size conditions. Lumen and mucus-associated microbiota compositions were analyzed by 16S rRNA Metabarcoding and differential analysis were further performed at the ASV level. Only stabilized points (from days 10 to 21) are represented. sPLS-DA analysis was performed to generate loading plots of the 15 most contributing ASVs between the luminal medium and mucin beads -all size confounded- (**a**) and between sizes -whatever the microenvironment- on component 1 (**d**) and 2 (**e**). Bars are colored according to the group in which the median abundance is maximal, which for each ASV, the relative abundancy indicated in grey. Species annotations are provided when a sequence identity percentage higher than 97% was identified using BLAST (given into bracket). Venn diagrams based on ASV repartition were also generated on both luminal medium (**b**) and mucin beads (**c**). ASV numbers and corresponding percentages (sequence number of ASV over total sequence number) are indicated.

**Fig. 4:**
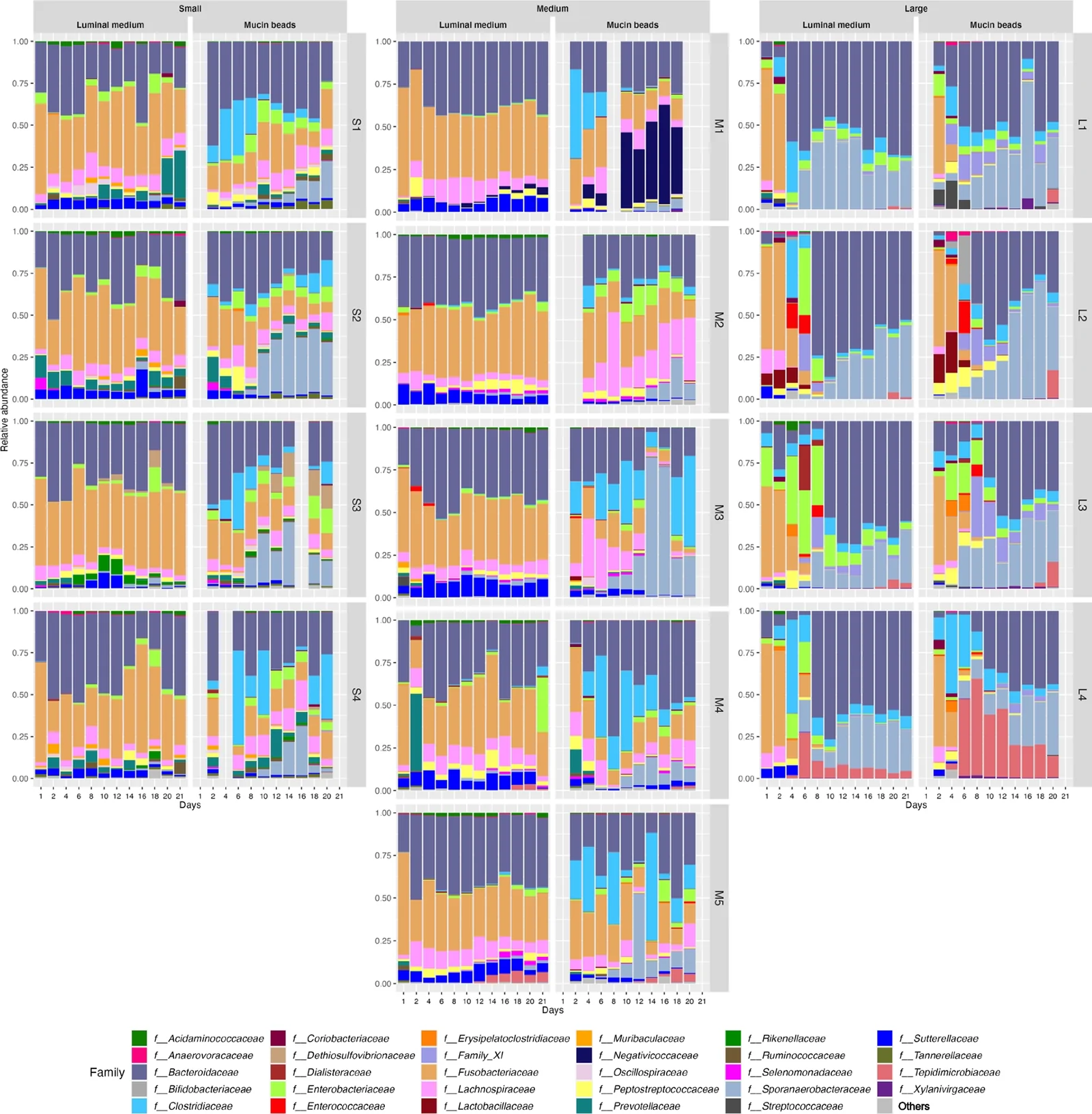
Microbiota composition of luminal medium and mucin beads at the family level. Fermentations were performed in the CANIM-ARCOL under three dog size conditions. Lumen and mucus-associated microbiota composition were analyzed by 16S rRNA metabarcoding at the family level. The most 30 abundant families are represented.

We also observed for the first time in a canine in vitro colon model, whatever the size conditions, a significant difference using a constrained RDA approach (*p* < 0.001) between lumen and mucus associated microbiota after stabilization, as shown by RDA analysis of microbiota composition (Fig. 2d). At the phylum level (Supplemental Fig. S4), for all dog sizes, higher abundances in *Firmicutes* together with lower amounts of *Fusobacteriota* were noticed in the mucosal microenvironment compared to the luminal one. Discriminant analysis showed that ASVs identified as *Ruminococcus* sp. and *Sporanaerobacter acetigenes* were enriched in the mucosal compartment (Fig. 3a). On the contrary, the luminal phase was enriched in *Fusobacterium mortiferum* and different ASVs identified as *Sutterella stercoricanis*. Of note, the mucus compartment was associated to a higher number of observed ASVs compared to the luminal phase, whatever the dog size (Fig. 2 a and b). The CANIM-ARCOL model also allows to capture inter-individual variabilities in microbial profiles. As an example, *Rikenellaceae* and *Dethiosulfovibrionaceae* were found only in the bioreactors inoculated with fecal sample from donor S3, while *Negativicocacceae* were observed only for M1 and *Anaerovoraceae* for L2 (Fig. 4). Regarding metabolic profiles, important methane levels (up 25 % at the end of fermentation) were observed in the small dogs (especially in bioreactors inoculated with stools from S2 and S3 dogs), while CH_4_ production did not exceed 0.1% in the medium and large size conditions. This was linked with the identification of methanogenic *Archaea* sequences (Supplemental Fig. S5) in both the lumen and mucus-associated microbiota of small dog bioreactors only. Whatever the colonic microenvironment, Archaea were identified as *Methanobrevibacter smithii* (data not shown).

### Size-specific colonic microbiota composition

**Fig. 5:**
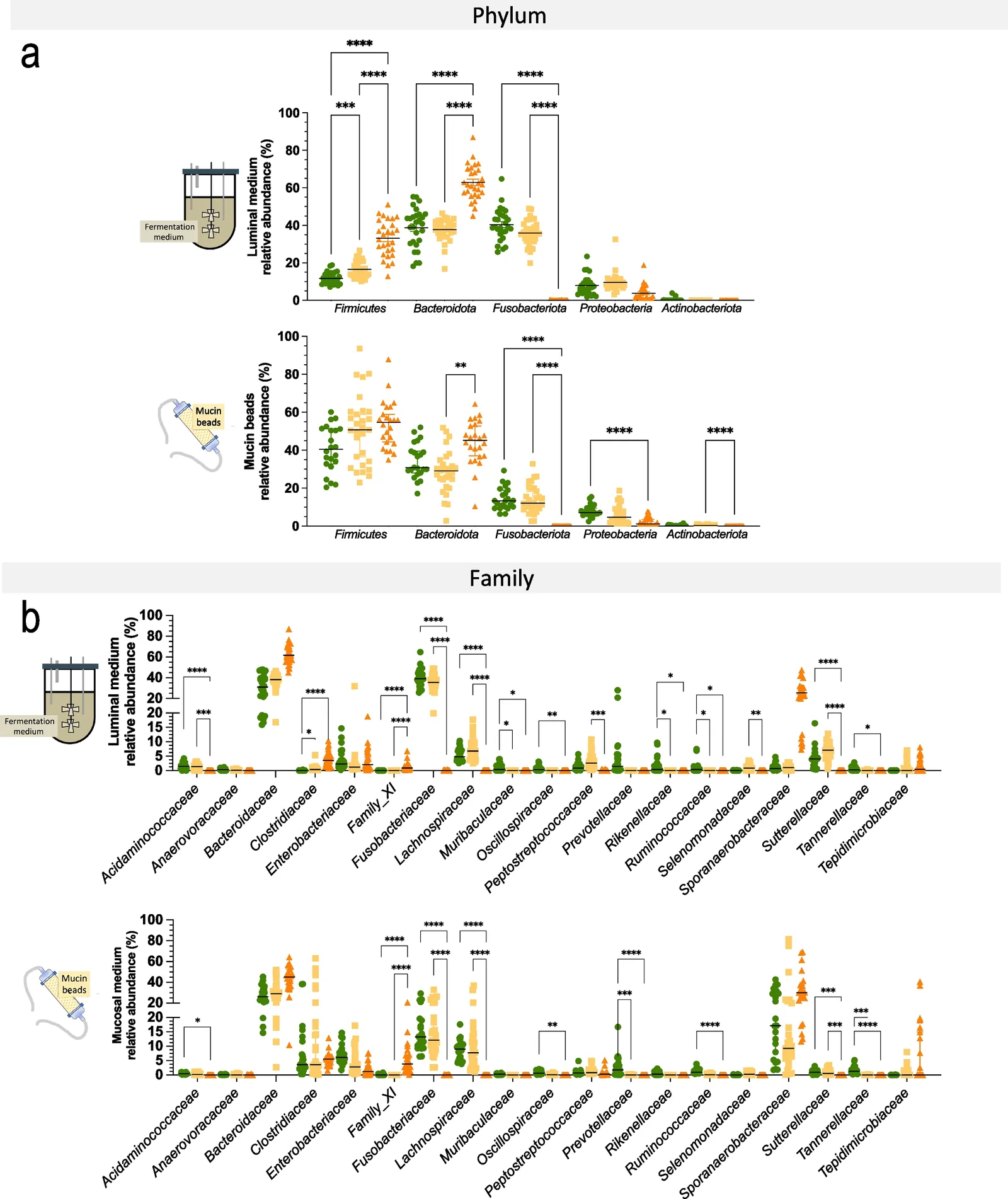
Impact of three dog sizes on microbiota composition at the phylum and family levels. Fermentations were performed in the CANIM-ARCOL under three dog size conditions. Lumen and mucus-associated microbiota composition were analyzed by 16S rRNA metabarcoding. Significant impacts of dog sizes on each bacterial population are indicated at the phylum (**a**) and family (**b**) levels (one-way ANOVA, double asterisks: *p* < 0.01; triple asterisks: *p* < 0.001; quadruple asterisks: *p* < 0.0001).

As mentioned before, three dog size conditions were reproduced in the CANIM-ARCOL, leading to clear different microbial profiles between sizes after stabilization (Fig. 4). At the phylum level (Fig. 5a), stabilized microbiota in the luminal fraction displayed from small to large size conditions an increase in *Firmicutes* (12, 17 and 33 % for small, medium, and large, respectively, *p* < 0.0001) and *Bacteroidota* (39, 38, and 63 %, respectively, *p* < 0.0001 between small/medium and large), whereas *Fusobacteriota* decreased with size (40, 36, and 0.1 %, respectively, *p* < 0.0001 between small/medium and large). At the family level (Fig. 5b), there were slight differences between small and medium sizes. However, *Lachnospiraceae*, *Prevotellaceae*, and *Sutterellaceae* decreased with size while *Clostridiaceae* and *Bacteroidaceae* increased from small to large group, with differences reaching significance between small/medium and large (Fig. 5b). In the mucosal compartment, at the phylum level (Fig 5a), stabilized mucus-associated microbiota was characterized by decreases with size in *Fusobacteriota* (13, 12 and 0 % for small, medium and large groups, respectively, *p* < 0.0001 between small/medium versus large), *Proteobacteria* (7, 5, and 1 %, respectively, *p* < 0.0001 between small versus large), and *Actinobacteriota* (0.2, 0.4, and 0 %, respectively, *p* < 0.0001 between medium versus large). At the family level (Fig. 5b), *Bacteroidaceae* increased with size (26, 29, and 45 % for small, medium and large size conditions, respectively, *p* < 0.0001 between small/medium versus large), while *Fusobacteriaceae* (13, 12, and 0%, respectively, *p* < 0.0001 between small/medium versus large) and *Lachnospiraceae* decreased (9, 8, and 0 %, respectively, *p* < 0.0001 between small/medium versus large). Venn analysis of 16S rRNA Metabarcoding data showed that only 23 ASVs from 10 different families were shared between the three size groups, representing 68.1 and 60.7% of total sequences in the luminal and mucosal compartments, respectively (Fig. 3b and c). The highest number of shared ASVs was found between small and medium group, whatever the compartment studied. Those results also displayed that a high number of low abundance-ASVs is constituting the size-specific microbiota, e.g., 316 ASVs (4.8 % abundance), 293 (6.7 %), and 39 (1 %) in the luminal phase of small, medium, and large size conditions, respectively. Discriminant analysis performed at the ASV level between the three size groups (whatever the colonic microenvironment, i.e., luminal or mucosal) revealed no significant difference between the small and medium groups and that the highest dissimilarities were observed between the small and large conditions (Fig. 3d). *Clostridium cochlearium* was enriched in the large group compared to the small one, while opposite trends were observed for *Fusobacterium* sp., *Bacteroides ovatus*, and *Bacteroides xylanisolvens*. Less discriminant differences were revealed by sPLS-DA (second component analysis; Fig. 3e) between medium and large groups with an enrichment in *Phocaeicola vulgatus*, *Fusobacterium mortiferum*, and *Sutterella stercoricanis* in the medium one. Mean alpha diversity indices (number of observed ASVs and Shannon index) were inversely correlated to canine size, in both lumen and mucus-associated microbiota (Fig. 2a and b). Redundancy analysis based on ASVs composition after stabilization demonstrated an obvious clustering by size (*p* = 0.001), stronger than that observed for donors (Fig. 2g). Moreover, weight (*p* = 0.001) was clearly identified as the main environmental parameters driving microbiota composition (Fig. 2d).

### Size-specific colonic microbiota activity

**Fig. 6:**
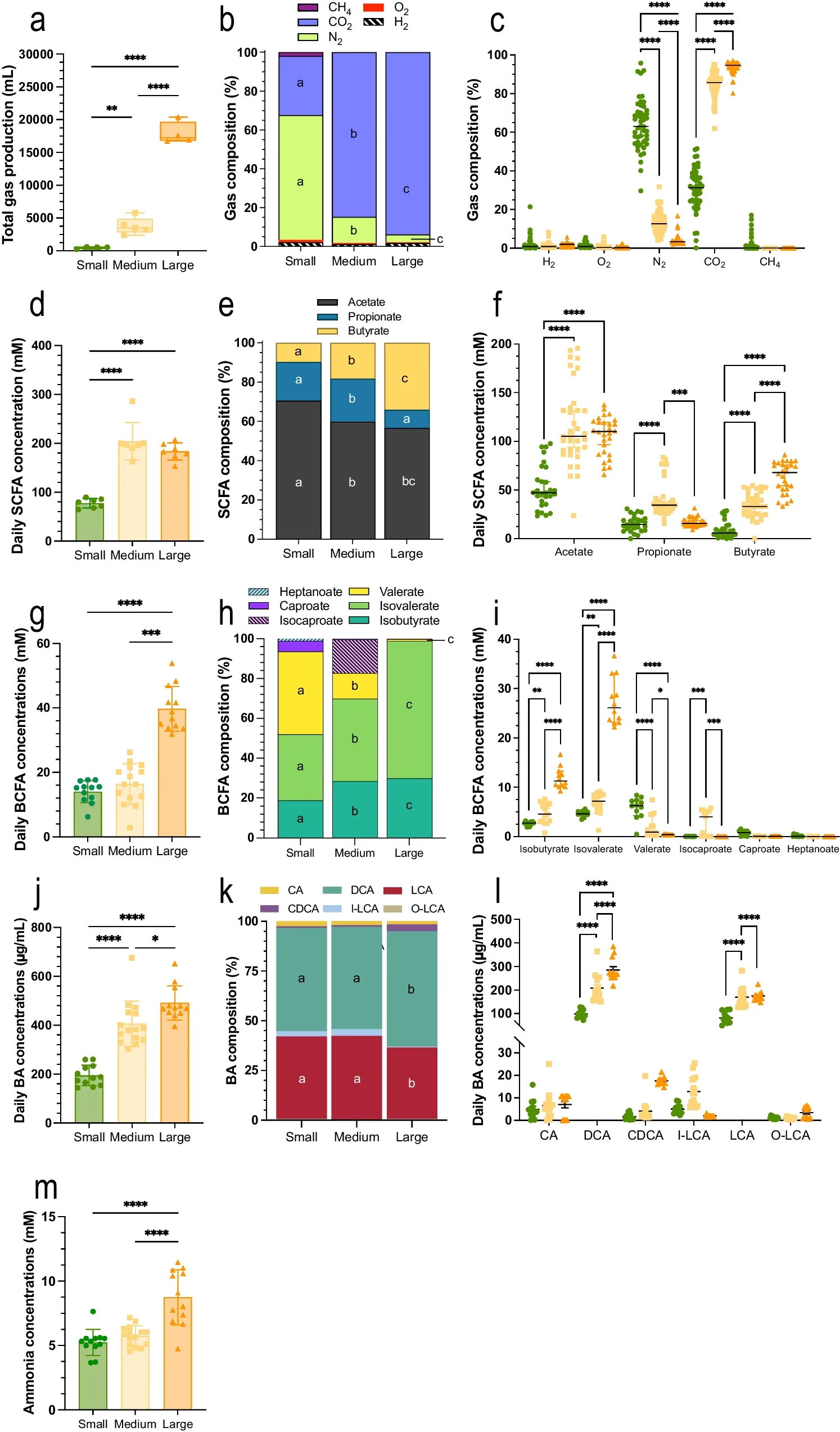
Impact of dog sizes on microbiota activity in the CANIM-ARCOL. Fermentations were performed in the CANIM-ARCOL under three dog size conditions. Samples were regularly collected from atmospheric phase to determine total gas production in milliliters (**a**) and gas composition in relative percentages depending on dog size conditions (**b**) or type of gas (**c**). The three main short-chain fatty acids (**d**,** e**, **f**), the six major branched chain fatty acids (**g**,** h**, **i**), ammonia (**j**), and main primary and secondary bile acids (**k**, **l**, **m**) were quantified in the luminal medium. Results are expressed as mean daily concentrations in mM ± SD (**d**, **f**, **g**, **i**, **k**, **m**) or relative percentages (**e**, **h**, **l**). All stabilized points (from 10 to 21 days) are represented for gas and SCFA, while only end points (from 18 to 21 days) are kept for BCFA, ammonia and BA. BA, bile acids; BA I, primary bile acids; BA II, secondary bile acids; BCFA, branched-chain fatty acids; CA, cholic acid; CDCA, chenodeoxycholic acid; CH_4_, methane; CO_2_, carbon dioxide; DCA, deoxycholic acid; H_2_, dihydrogen; I-LCA, Isoallo-3-ketocholate; LCA, lithocholic acid; N_2_, nitrogen; O-LCA, 3-oxolithocholic/dehydrolithocholic acid; O_2_, dioxygen; SCFA, short-chain fatty acids. Statistical differences are indicated by letters or single asterisk: *p* < 0.05, double asterisks: *p* < 0.01; triple asterisks: *p* < 0.001; quadruple asterisks: *p* < 0.0001 (one-way ANOVA).

Total gas production significantly increased (*p* < 0.01) with size (Fig. 6a), with a mean total production over the stabilized phase (10–21 days) of 475, 3775, and 17,920 mL for the small, medium, and large groups, respectively. This was associated to clear different gas profiles between the three size groups (Fig. 6b and c). CO_2_ percentages significantly increased (*p* < 0.0001) with sizes, ranging from 30 to 94 % from the small to large conditions. Methane and oxygen tended to be more abundant in the small size group, with average oxygen percentages of 1.3, 0.6, and 0.4 % and methane percentages of 1.9, 0.01, and 0.03 %, for the small, medium, and large groups, respectively.

A clear size effect on SCFA and BCFA production was also shown (Fig. 6d and g). Daily SCFA concentration in the stabilized phase (Fig. 6d) increased with size from 83 mM for small to 179 mM for large, with differences reaching significance for small versus medium and small versus large conditions (*p* < 0.0001). Similarly, daily BCFA production (Fig. 6g) was positively correlated to canine size with 14, 16, and 40 mM for small, medium, and large dogs, respectively (*p* < 0.0001 for small versus large and *p* < 0.001 for medium versus large). Mean SCFA profiles also differed between groups with a significant increase in the ratio and concentration of butyrate (*p* < 0.001) from small to large dogs (Fig. 6e and f). The percentages of acetate and propionate (but not their concentrations) also decreased with size. BCFA profiles were also widely impacted by size effect. Caproate and heptanoate were found only under small size condition, while isocaproate was medium size-specific (Fig. 6h). Isobutyrate and isovalerate ratios and concentrations significantly (*p* < 0.01) increased with canine size, whereas valerate decreased (Fig. 6h and i). RDA analysis of sequencing data implemented with SCFA concentrations indicated that luminal composition of medium dog size is mostly correlated to propionate level whereas luminal composition of large dog size is mostly correlated to butyrate (Fig. 2g).

Daily bile acid concentrations significantly increased with size (*p* < 0.05), with around 200 µg/mL in the fermentation medium from small bioreactors versus 400 and 500 µg/mL under medium and large conditions, respectively (Fig. 6j). Associated profiles also significantly differed with dog size, with a significant decrease of LCA percentage together with a rise in DCA in large versus small and medium conditions (Fig. 6k). In addition, in large bioreactors, percentages of CDCA tended to be higher and those of I-LCA lower compared to the two other conditions. Lastly, both DCA and LCA concentrations increased significantly (*p* < 0.0001) with dog size (Fig. 6l). Daily ammonia concentration (Fig. 6m) in the luminal compartment increased with size (*p* < 0.0001 for small versus large and medium versus large), with a mean total value over the stabilized phase (10–21 days) of 5.2, 5.7, and 8.7 mM for small, medium, and large dogs, respectively.

## Discussion

In accordance with the “3Rs” rules (adapted from Russel and Burch 1959) which prone the reduction of animal use and the development of in vitro alternative strategies, the main objective of this study was to develop and validate through in vitro-in vivo comparisons the first model reproducing the canine colonic ecosystem adapted to three dog sizes, the CANIM-ARCOL. This was achieved thanks to a wide literature review (150 publications) we previously performed on canine colonic physicochemical (pH and transit time), nutritional (composition of simulated ileal effluents including nutrients and bile acids), and microbial (gut microbes’ composition and functionalities) parameters (Deschamps et al. 2022b). Up to now, most of the systems (8 out of 10) developed to reproduce the canine colonic environment are static batch models (Sunvold et al. 1995; Tzortzis et al. 2004; Bosch et al. 2008; Cutrignelli et al. 2009; Panasevich et al. 2013; Vierbaum et al. 2019; Duysburgh et al. 2020; Van den Abbeele et al. 2020; Oba et al. 2020). Compared to CANIM-ARCOL, such systems are much more simplified related to physiological conditions, excluding digestive regionalization and dynamism (Table 2). Moreover, experiments are limited in time (up to 72 h), allowing only short-term analysis and preventing in vitro simulation of chronic ingestion of any compound of interest. The only dynamic model currently available with a similar level of complexity is the M-SCIME (Verstrepen et al. 2021). Compared to this system, besides reproducing size-related conditions, CANIM-ARCOL exhibits the unique feature to maintain anaerobiosis by the sole activity of resident microbiota, allowing an interesting follow up of atmospheric gases. However, it does not simulate like the M-SCIME the three colonic parts nor passive absorption of fermentation products which is a key feature in gut homeostasis (Weber et al. 2004). For the first time, even if it was limited by the scarcity of information (in medium dogs only), we also compared our in vitro data to in vivo data obtained from the canine large intestine. In all other available models, if performed, validation was only based on data from canine fecal samples. Together with the batch model of Oba et al. (2020) and M-SCIME (Verstrepen et al. 2021), our model is one of the rare systems to distinguish the luminal from the mucus-associated microbiota, aiming to recreate more physiologically the different colonic microenvironments. Here, the mucin-compartment was filled with beads made with mucin from porcine stomach, the only source yet commercialized. Even if using canine colonic mucins will be more relevant, this option is hampered by obvious technical, societal, and regulatory limitations. Anyhow, MUC-5AC and MUC-5B found in pig mucins are also the most represented glycoproteins in canine large intestine mucus (Dubbelboer et al. 2022). As previously observed in human and pig in vitro studies, adding a mucosal compartment allowed to capture a higher bacterial diversity from the fecal microbiome (Van den Abbeele et al. 2009; Deschamps et al. 2020; Van Herreweghen et al. 2021; Gresse et al. 2021; Verstrepen et al. 2021). For each size condition and donor, this compartment exhibited a higher number of observed ASVs compared to the luminal one and is particularly efficient to preserve bacteria from the *Firmicutes* phylum, such as *Ruminococcaceae* and *Clostridiaceae* (Maru et al. 2018). It was particularly helpful in maintaining rare taxa from *Tannerellaceae* or *Ruminococcaceae* families, in line with their mucin-degrading bacteria status (Bell et al. 2008), certainly by providing specific nutritional niches. Lastly, as previously described for the M-SCIME, making use of different fecal samples enabled our new in vitro model to capture the interindividual variability in colonic microbiome. Keeping variability associated to an individual or its breed is of high importance, since it is acknowledged as an important feature in canine gut microbiota and health (Oswald et al. 2015).

**Table 2:**
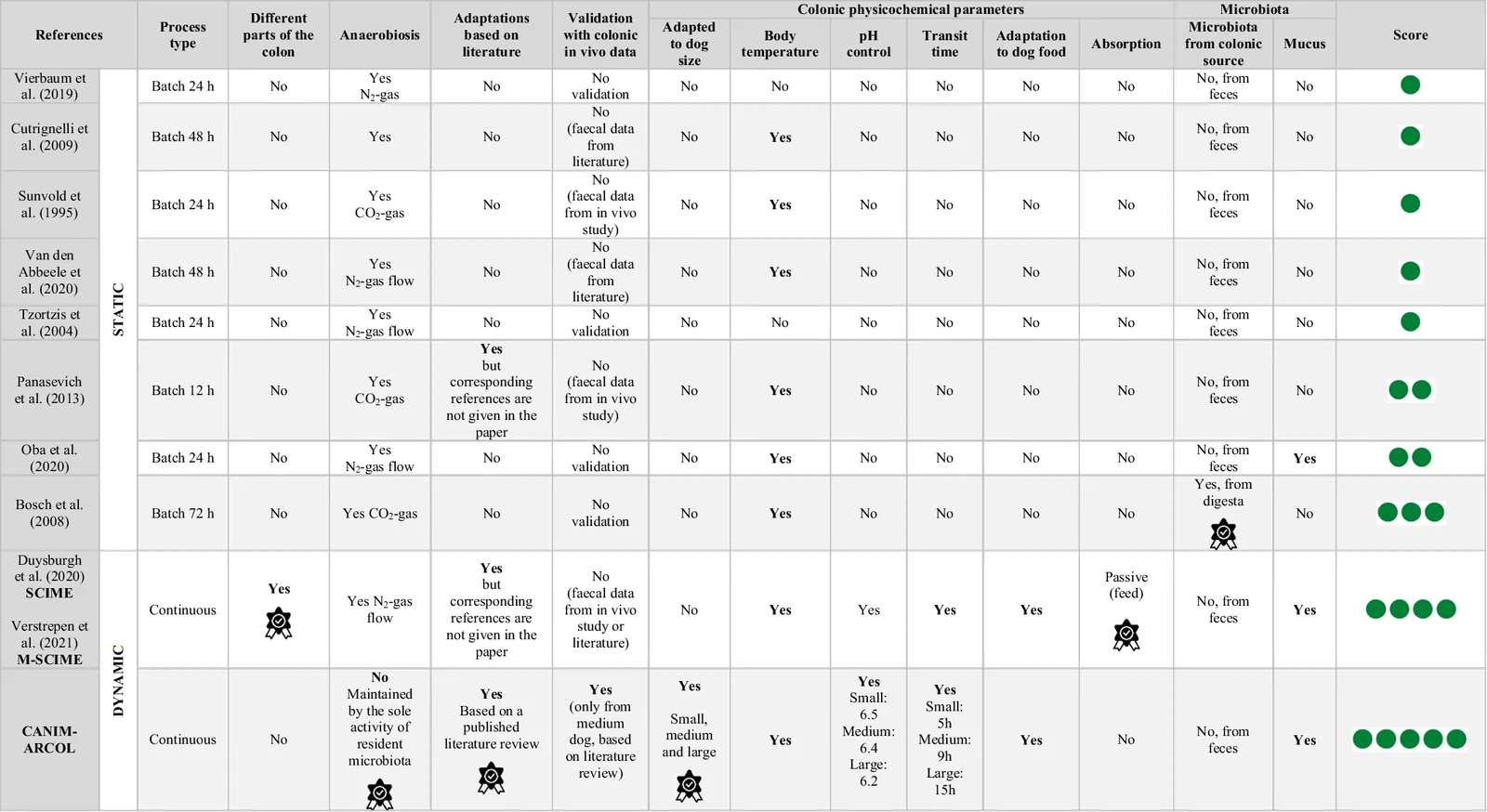
Comparison of the CANIM-ARCOL model and other currently available models of the dog large intestine

As previously mentioned, a major advance associated with this in vitro gut model development is the possibility to reproduce and discriminate digestive conditions associated to different dog sizes (i.e., “small” under 10 kg, “medium” from 10 to 30 kg, and “large” over 30 kg). To ensure the relevance of the newly developed size-related colonic model, our in vitro results were challenged with in vivo data. Due to the paucity of information on canine colonic microbiota (only two studies performed in medium dogs, none with medium and large dogs), in vitro-in vivo comparisons on colonic data were only made on medium dog size (Table 3). In addition, since there is no information on colonic microbiota activity in vivo, such analysis was only based on microbiota composition. In both luminal and mucosal compartments, the CANIM-ARCOL model allowed to maintain in vitro the dominant bacterial phyla inhabiting the canine colon (Table 3), i.e., *Firmicutes*, *Bacteroidota*, *Fusobacteriota*, and *Proteobacteria*, based on data collected from one study on intraluminal colonic content (Suchodolski et al. 2008) and other one on colonic biopsies (Honneffer et al. 2017). Interestingly, our in vitro system preserved 27 of the 31 families detected in vivo, apart from *Prevotellaceae*, *Streptococcaceae*, *Turicibacteraceae*, and *Veillonellaceae*. Of note, these four populations were only found in colonic biopsies, but not mentioned in the study on intraluminal colonic content (Suchodolski et al. 2008; Honneffer et al. 2017).

**Table 3:**
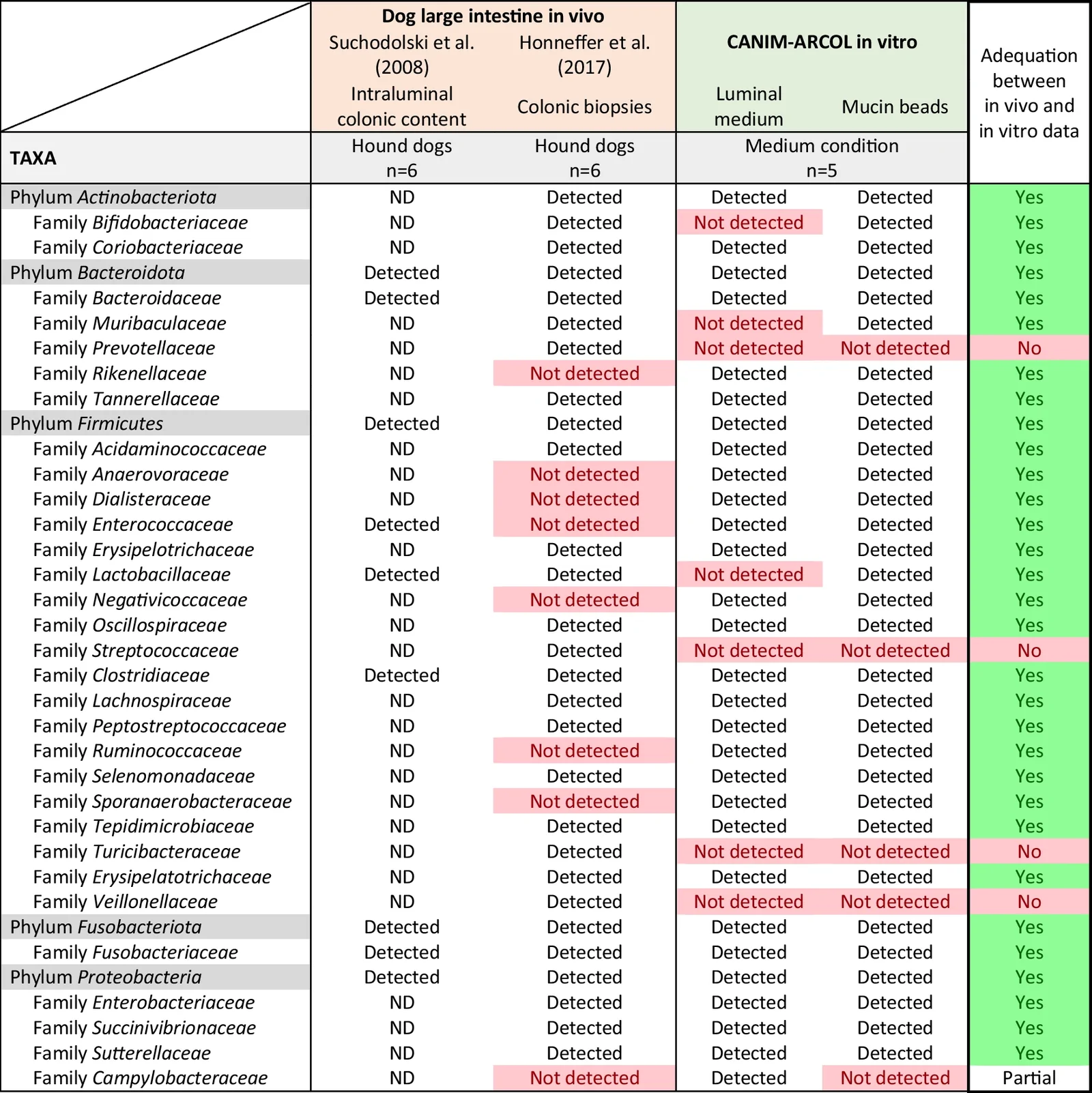
Comparison of main bacterial populations found in the CANIM-ARCOL model under medium size conditions and *in vivo* in the large intestine of medium size dogs

Similarity or discordance between in vitro and in vivo data are indicated by a green and red color code, respectively

ND, not determined

The second level of in vitro-in vivo comparisons was made on the three dog sizes conditions between our in vitro colonic results and data from dog fecal samples collected in a previous literature review from us (Deschamps et al. 2022b; Table 4). The impact of dog sizes on in vitro results was in line with in vivo data for four out of the five main bacterial phyla (*Actinobacteria*, *Bacteroidota*, *Firmicutes*, and *Proteobacteria*), but not *Fusobacteriota*. In the present study, this population almost disappeared from large bioreactors, while an abundance around 6 % was assumed in fecal samples from large dogs by two in vivo studies (Sandri et al. 2016; Hullar et al. 2018). This inhibition might be related to the high levels of BCFA found under colonic large size conditions in vitro, and not observed in vivo, as explained later. In our study, microbial alpha-diversity was inversely correlated with dog size. This is not fully in accordance with in vivo funding which indicates the highest diversity for medium dog. However, both in vitro and in vivo results associated the lowest microbial diversity with large dog size condition (Sandri et al. 2016). This might be related to the lower amount of soluble fibers (compared to insoluble ones) introduced in the nutritive medium under large dog conditions in vitro, in accordance with in-field recommendations in this population with particular digestive sensitivity (Weber et al. 2017). Increasing the amount of soluble fibers has already been associated with a higher fecal diversity in dogs (Biagi et al. 2008; Chen et al. 2019). In any event, alpha diversity indexes measured in vitro in large bioreactors looked low compared to the physiological situation (Sandri et al. 2016). This may be due to the accumulation of SCFA, BCFA, and/or ammonia in the bioreactors under large size condition which can inhibit some bacterial population and favor other ones reducing alpha-diversity value (Cui et al. 2021). Lastly, of particular interest, methanogens *Archaea*, represented by *M. smithii*, were found in our in vitro model, but under small size conditions only. This constitutes the first description of methanogens *Archaea* maintenance under in vitro canine digestive conditions. In vivo, this population is also very poorly described since only one study reported their presence in medium dog stools (Deng and Swanson 2015), and evidently, no data is available on the effect of dog size. Such *Archaea* distribution in bioreactors cannot be related to initial load in fecal sample, this population being detected in two medium dog fecal samples only. *Archaea* occurrence under small dog size condition in vitro is in contradiction with previous funding in human showing a positive correlation between their abundance and prolonged gastrointestinal transit time (Gaci et al. 2014). One hypothesis would be related to lower isovalerate concentrations in small size conditions compared to medium and large ones, in accordance with Liu and collaborators that previously showed that total methanogens were linearly reduced with increasing isovalerate supplementation in steers rumen (Liu et al. 2014).

**Table 4:**
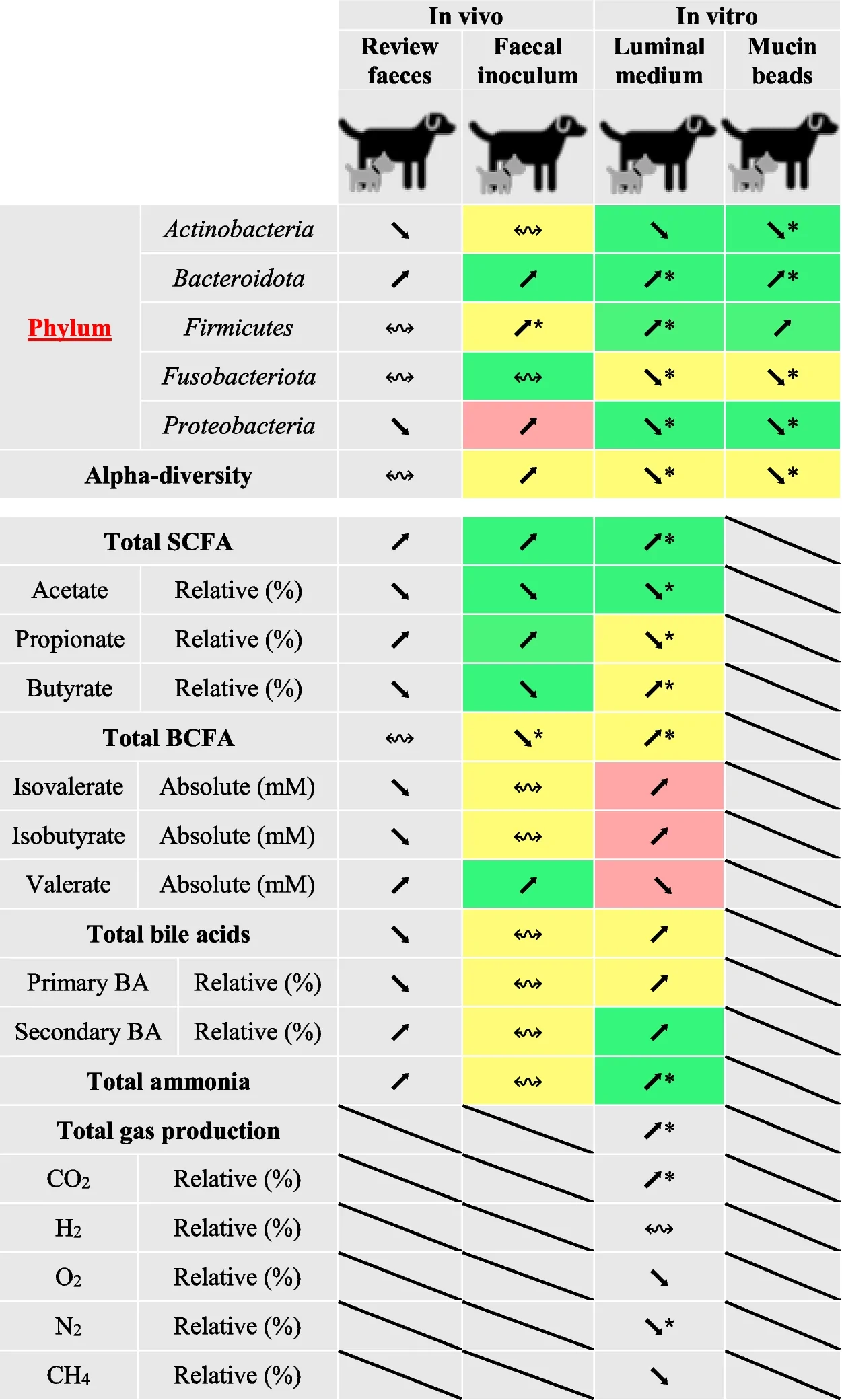
In vitro-in vivo comparisons of dog size impact on gut microbial composition and activity based on fecal data.  This table makes a comparison between in vitro data in CANIM-ARCOL and in vivo data in dog fecal samples, gathered from our previous literature review (Deschamps et al. 2022b) or issued from the analysis of stool samples used in this study to inoculate the bioreactors (Supplemental Fig. S1 ). Inclusive parameters for in vivo data are the following: healthy adult dogs, fed with dry food, and classified according to their size in small (body weight under 10 kg), medium (from 10 to 30 kg), or large dogs (over 30 kg). Green box: in vitro data obtained in CANIM-ARCOL are in line with canine in vivo data; yellow: no clear conclusion can be found due to lack of in vivo data; red: in vivo and in vitro data are contradictory; ⬊: less abundant from small to large conditions; ⬈: more abundant from small to large conditions; ↭: no clear change with size conditions. Lack of data is symbolized by a diagonal black line.  *BA* bile acids, *BCFA* branched chain fatty acids, *CA* cholic acid, *CDCA* chenodeoxycholic acid, *DCA* deoxycholic acid, *LCA* lithocholic acid, *SCFA* short-chain fatty acids. * Significant variation (*p* < 0.05)

In an original way, this in vitro model development also provided for the first time plenty of data on colonic microbiota activities related to dog size conditions (Table 4), with measurement of main end-fermentation products such as atmospheric gases (allowed by the lack of flushing in bioreactors with N_2_ or CO_2_), SCFA, BCFA, ammonia and bile acid dehydroxylation activities through primary and secondary bile acid dosage. In the canine large intestine, complex polysaccharides are degraded into monosaccharides leading to gas and SCFA production. In the present study, total gas production increased with dog size, in line with a higher microbial fermentation activity in large dogs compared to smaller sizes (Weber et al. 2004). This study provides the first set of data on gas profiles under canine colonic conditions since there is no information in dogs. Higher O_2_ and lower CO_2_ levels in the atmospheric phase of small bioreactors are directly related to a lower microbial fermentation activity. More remnant H_2_ in large bioreactors also correlates with a higher abundance of H_2_-producers bacteria such as *Bacteroides* and *Clostridium* (Wolf et al. 2016). In addition, high proportion of CH_4_ under small size condition is perfectly correlated with the presence of *M. smithii*, as previously mentioned. Regarding SCFA, total concentrations in vitro was positively associated with dog size, in accordance with in vivo data in fecal samples and the higher transit time in large dogs favoring microbial fermentation (Weber et al. 2004). The effect of dog size on acetate relative percentage was in line with in vivo data (Cutrignelli et al. 2009; Beloshapka et al. 2014; Sandri et al. 2016; Paßlack et al. 2021; Meineri et al. 2022), but not that of butyrate and propionate. The highest concentrations in butyrate were observed in large bioreactors, certainly linked to the presence of *Clostridiaceae* in the luminal medium, known to be involved in carnivorous butyrate production pathway (Vital et al. 2015). BCFA and ammonia result from metabolization by microbiota of undigested dietary proteins (Davila et al. 2013). Total BCFA concentrations increased with dog size in vitro, while no clear conclusion was provided by in vivo studies (Deschamps et al. 2022b). Besides, opposite trends were observed between our in vitro colonic results and in vivo data in dog stools regarding isovalerate, isobutyrate and valerate concentrations (Cutrignelli et al. 2009; Beloshapka et al. 2014; Sandri et al. 2016). Regarding ammonia, also resulting from protein fermentation, in vitro concentrations increased from small to medium size conditions, accordingly with data in dog stools (Deschamps et al. 2022b). Such a rise might be associated to the bloom of *Clostridiaceae* and *Sporanaerobacteraceae* observed in large bioreactors (Hardy et al. 2021). Lastly, we followed bile acid dehydroxylation of primary (CA and CDCA) into secondary bile acids (LCA and DCA) by colonic microbiota in vitro. Such process was identified as a key health marker disturbed in canine diseases such as antibiotic-induced dysbiosis (Whittemore et al. 2021), chronic enteropathies (Guard et al. 2019), or obesity (Apper et al. 2020). Total bile acid concentrations increased in vitro with dog size, while opposite trends seemed to be observed in dog stools, in the only available study (Guard et al. 2019). Our study described for the first time that deconjugation of primary bile acids (supplied by the nutritive medium; Table 1) into secondary bile acids efficiently occurs in an in vitro canine model. This certainly results from the activity of bile acid metabolizing bacteria such as *Bacteroides*, *Clostridium*, and *Lactobacillus* (Rowland et al. 2018). In addition, relative percentages of secondary bile acids raised with dog size, consistent with in vivo data in fecal samples (Guard et al. 2019).

To conclude, for the first time, we set up a new size-related in vitro model of the dog large intestine, the CANIM-ARCOL. This model was well validated through in vitro-in vivo comparisons for the medium size condition and a discriminant size-effect was reproduced. The model development also provided useful data regarding mucus-associated microbiome and microbiota metabolic activities under canine colonic simulated conditions. However, the validation of small and large size bioreactors suffered from the lack of colonic in vivo data and the paucity of fecal ones. Recent developments of non-invasive methods like wireless motility capsules (Warrit et al. 2017) or medical device aiming to collect microbiota during gastrointestinal transit open new avenues to fill these scientific and technological gaps. This in vitro model represents a powerful platform to study the fate of food and veterinary products in the canine digestive environment, help to elucidate their mechanisms of action in relation with colonic microbiota, and promote innovation in these fields. Of particular interest, CANIM-ARCOL allows to study the bilateral interactions between gut microbiota and any positive (e.g., nutrients, fiber, pre-, pro or postbiotics, drugs) or deleterious compounds (e.g., pathogens, pollutants, mycotoxins) crossing the large intestine in dogs, without confounding host effect. Therefore, valuable information regarding not only the impact of those compounds on microbiota composition and activities but also, reversely, their metabolization by microbiota can be obtained. This model will also help to move toward personalized nutrition or medication, by capturing interindividual or breed variabilities in gut microbiome and considering dog body weight (Oswald et al. 2015; You and Kim 2021). Further developments would include the coupling of CANIM-ARCOL model with intestinal or immune cells to integrate host interactions and the adaptation of the model to diseased situations, such as antibiotic-induced dysbiosis (Igarashi et al. 2014), chronic enteropathies (AlShawaqfeh et al. 2017), or obesity (Apper et al. 2020).

## Supplementary Information

Below is the link to the electronic supplementary material.

Supplementary file1 (PDF 3013 KB)

## Data Availability

Data transparency. Raw Metabarcoding data are available at NCBI under the Sequence Read Archive database with accession number no. PRJNA955438.
