# Supplementary material for: Canine Mucosal Artificial Colon: development of a new colonic in vitro model adapted to dog sizes
Source: Appl Microbiol Biotechnol. 2024 Jan 23;108:166. doi: 10.1007/s00253-023-12987-2 (PMC10806056; doi:10.1007/s00253-023-12987-2)
Supplement: Supplementary file 1 [file 253_2023_12987_MOESM1_ESM.pdf]

Applied Microbiology and Biotechnology

Canine Mucosal Artificial Colon: development of a new colonic in vitro model adapted to dog sizes

Charlotte Deschamps[1,2], Sylvain Denis[1], Delphine Humbert[3], Nathalie Priymenko[4], Sandrine Chalancon[1], Jana De Bodt[5], Tom Van de Wiele[5], Ignacio Ipharraguerre[6], Inma Alvarez-Acero[7], Caroline Achard[2], Emmanuelle Apper[2], Stéphanie Blanquet-Diot[1*]

[1]Université Clermont Auvergne, UMR 454 MEDIS UCA-INRAE, F-63000 Clermont-Ferrand, France. [2]Lallemand Animal Nutrition, F-31702 Blagnac Cedex, France. [3]Dômes Pharma, 63430 Pont-du-Château, France.
[4]University of Toulouse, Toxalim (Research Center in Food Toxicology), INRAE, ENVT, INP-Purpan, UPS, 31000 Toulouse, France. [5]Center for Microbial Ecology and Technology (CMET), Faculty of Bioscience Engineering, Ghent University, Ghent, Belgium. [6]Institute of Human Nutrition and Food Science, University of Kiel, Germany. [7]Institute of Food Science, Technology and Nutrition, Spanish National Research Council (ICTAN-CSIC), Madrid, Spain.

***Corresponding address**:
Stéphanie Blanquet-Diot
UMR 454 MEDIS
University Clermont Auvergne, INRAE
28, place Henri Dunant
63000 Clermont-Ferrand, France
(+33473178390, stephanie.blanquet@uca.fr)

Supplementary tables

Supplemental Table S1 Characteristics of healthy adult dogs included in the study and ranked by small, medium and large dog sizes

M: male, F: female, BCS: body condition score (ranging from 1 -very thin- to 5 -obese-, 3 corresponding to ideal weight)

Size	Dog_id	Breed	Sex	Sterilization	Age (years)	BCS	Weight (kg)	Garden access	Feed
**Small**	S1	Shih Tzu	M	No	6	3	5	No	Dry
	S2	Chihuahua	F	No	7	3	1,9	Yes	Dry
	S3	Cavalier King Charles	F	No	5.5	3	8	No	Dry
	S4	Spitz	F	Yes	2	3	4	No	Dry
**Medium**	M1	Border collie x Beauceron	M	Yes	2.5	3	30	Yes	Dry
	M2	French bulldogged	F	Yes	6	3	13	Yes	Dry
	M3	Border collie	M	Yes	6	3	18	Yes	Dry
	M4	Flat coater Retriever	F	No	2.5	3	28	Yes	Dry
	M5	Golden Retriever	F	No	6.5	3	29	Yes	Dry
**Large**	L1	Saint-Bernard	M	No	6	3	80	Yes	Dry
	L2	Beauceron	F	Yes	3	3	40	Yes	Dry
	L3	Leonberg	F	No	3	3	52	Yes	Dry
	L4	German Shepherd	M	No	5	3	42	Yes	Dry

Supplemental Table S2 Primers used for qPCR and 16S rRNA Metabarcoding analysis

Primer names	Sequence 5′-3′	Target	Annealing temperature (°C)	References
**qPCR primers**				
BAC338R	ACTCCTACGGGAGGCAG	Total *Bacteria*	58	Yu et al. (2005)
BAC516F	GTATTACCGCGGCTGCTG			
**Metabarcoding primers**				
V3_F357_N	CCTACGGGNGGCWGCAG	*Bacteria*	-	Klindworth et al. (2013)
V4_R805	GACTACHVGGGTATCTAATCC			
Arch349F	GYGCASCAGKCGMGAAW	*Archaea*	-	Takai and Horikoshi (2000)
Arch806R	5GGACTACVSGGGTATCTAAT			

Supplementary figures

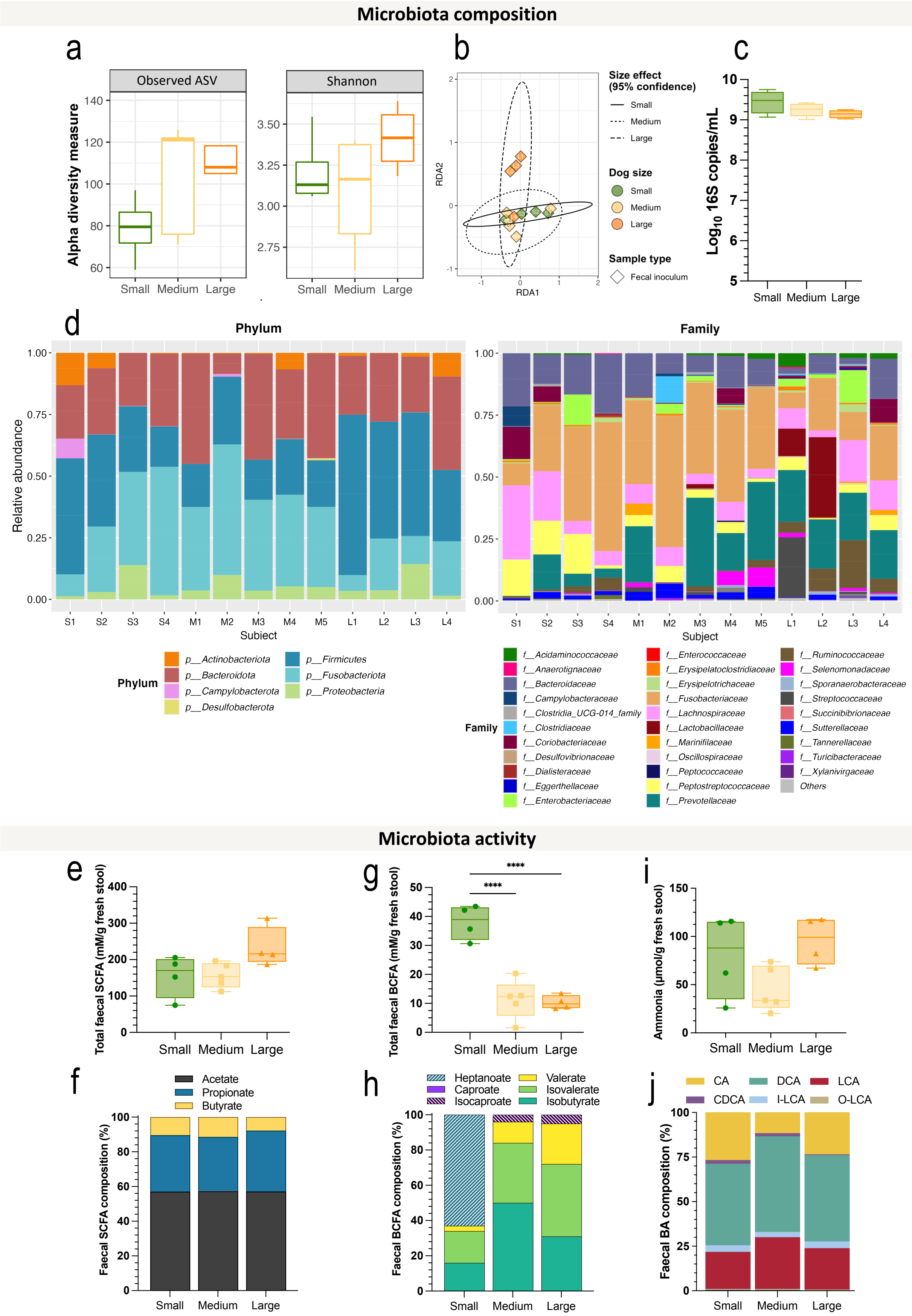

Supplemental Fig. S1 Stool characterization for each canine donor

Stool samples were collected from 13 healthy dogs (n=4 small in green, n=5 medium in yellow and n=6 large in orange). Microbiota composition was analyzed by 16S Metabarcoding. Diversity indexes were calculated based on ASV table. α-diversity (observed ASVs and Shannon Index) is represented as box plots for each dog size (**a**). Redundancy analysis (RDA) two-dimension plot visualizations reported bacterial community β-diversity, showing the effects of dog size (**b**), numbers refer to dog_id mentioned in **Table S1**. Total bacteria were also determined by quantitative-PCR and plotted as boxplots (**c**). Bacterial abundances are shown at the phylum and family levels (**d**). The three main short-chain fatty acids (**e**, **f**), the six major branched-chain fatty acids (**g**, **h**), ammonia (**i**) and main primary and secondary bile acids (**j**) were quantified in the stool samples. Results are expressed as mean daily concentrations in mM/g of fresh stool ± SD (**e, g**, **i**) or relative percentages (**f**, **h**, **j**). BCFA: branched-chain fatty acids, CA: cholic acid, CDCA: chenodeoxycholic acid, DCA: deoxycholic acid, I-LCA: isoallo-3-ketocholate, LCA: lithocholic acid, O-LCA: 3-oxolithocholic/dehydrolithocholic acid, SCFA: short-chain fatty acids. Statistical differences are indicated by ****: $p<0.0001$ (ANOVA one-way).

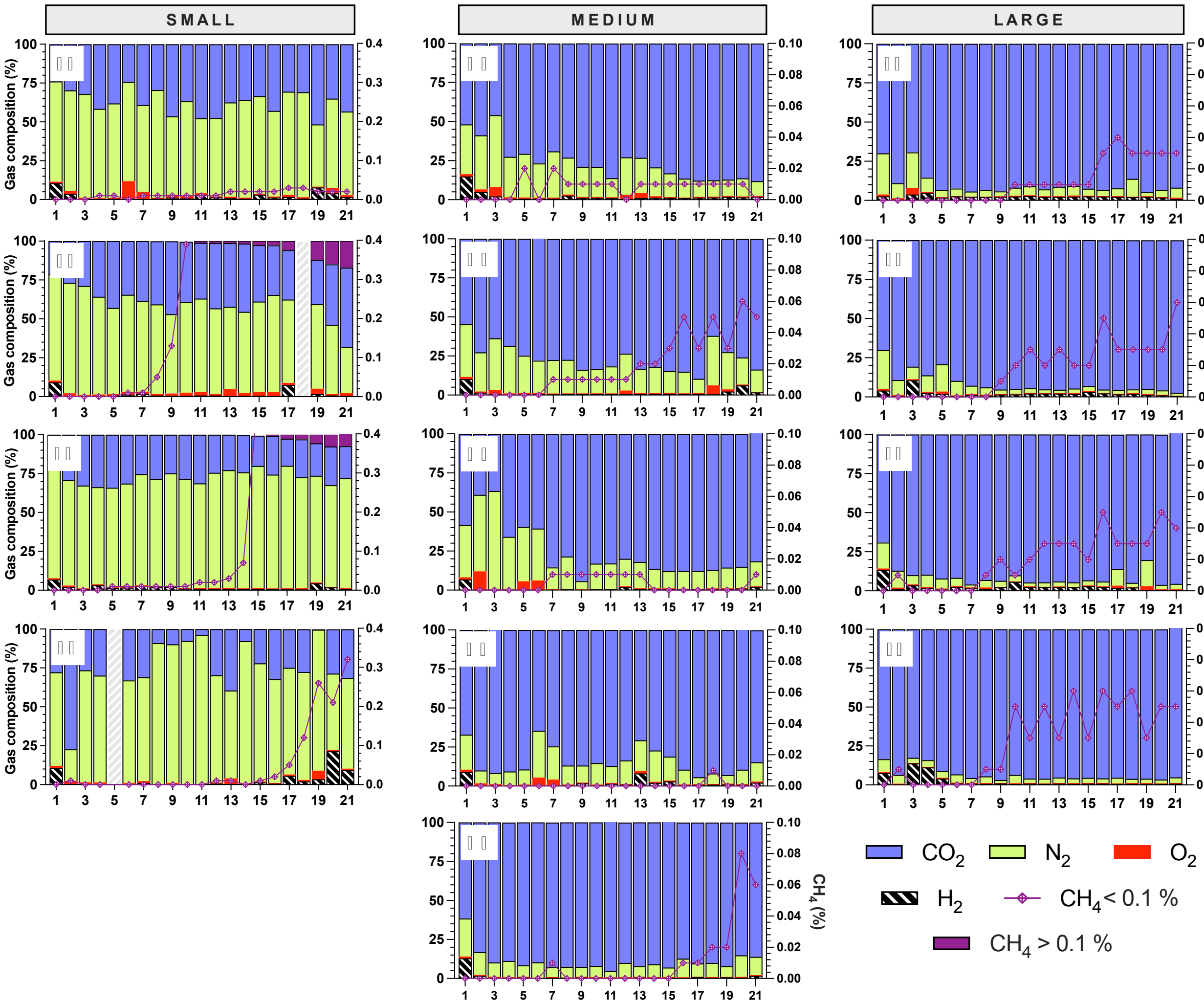

Supplemental Fig. S2 Impact of three dog sizes on gas composition in the CANIM-ARCOL at the individual level

Fermentations were performed in the CANIM-ARCOL under three dog size conditions, when bioreactors were inoculated with fecal samples from 13 healthy dogs (n=4 small "S", n=5 medium "M" and n=6 large "L"). Atmospheric phase of bioreactors was sampled daily to monitor gas composition throughout fermentations. Results are expressed as relative percentages of main gases. CH_4: methane, CO_2: carbon dioxide, H_2: dihydrogen, N_2: nitrogen, O_2: dioxygen.

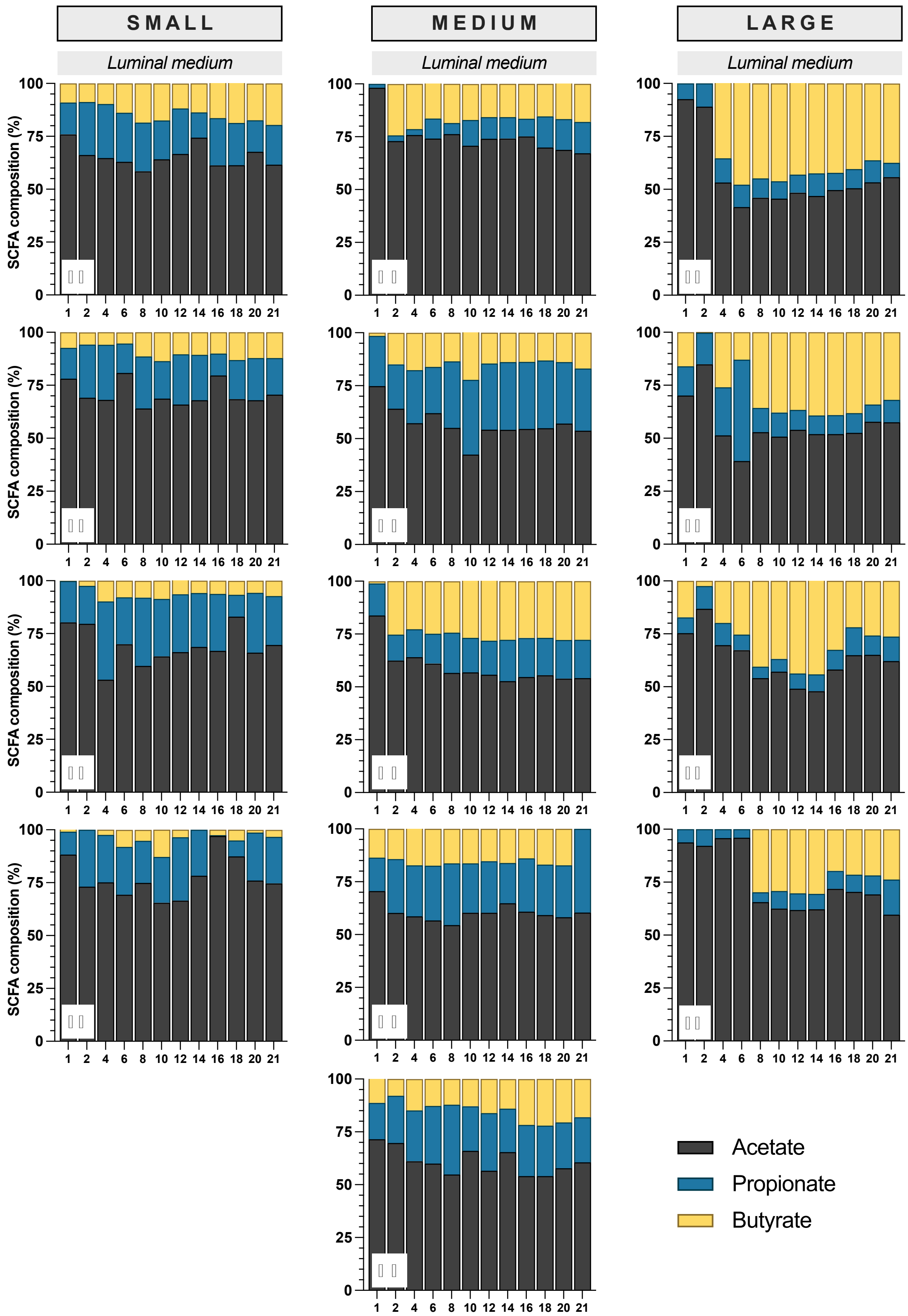

Supplemental Fig. S3 Impact of three dog sizes on SCFA composition in the CANIM-ARCOL at the individual level

Fermentations were performed in the CANIM-ARCOL under three dog size conditions, when bioreactors were inoculated with fecal samples from 13 healthy dogs (n=4 small "S", n=5 medium "M" and n=6 large "L"). Luminal medium of bioreactors was sampled daily to monitor short chain fatty acid composition throughout fermentations. Results are expressed as relative percentages of main SCFA (i.e. acetate, propionate and butyrate).

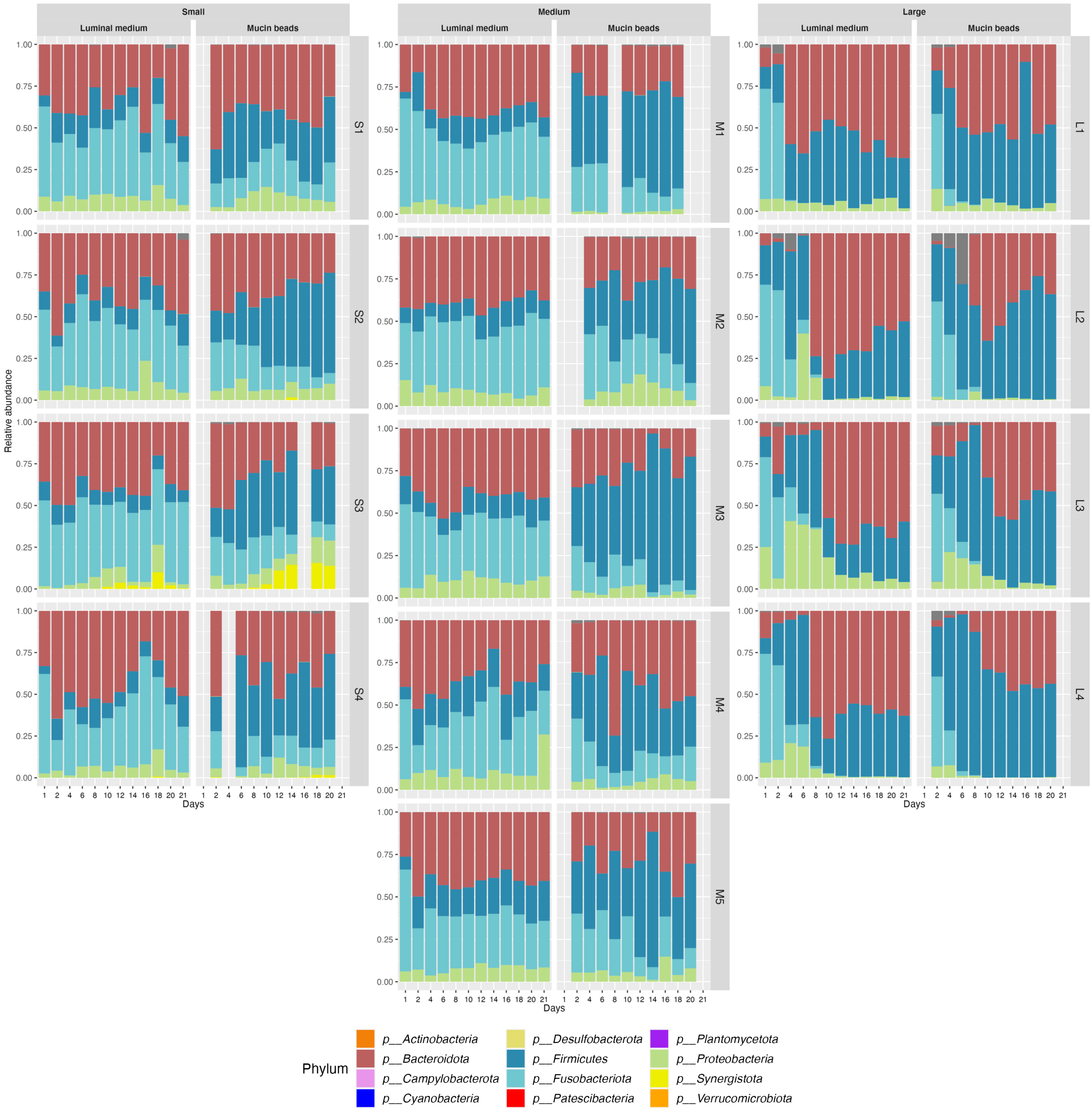

Supplemental Fig. S4 Impact of three dog sizes on microbiota composition in the CANIM-ARCOL at the individual level Fermentations were performed in the CANIM-ARCOL under three dog size conditions, when bioreactors were inoculated with fecal samples from 13 healthy dogs (n=4 small "S", n=5 medium "M" and n=6 large "L"). Luminal medium of bioreactors and mucin beads were sampled daily to monitor microbiota composition at the phylum level. Results are expressed as relative abundances of main phyla.

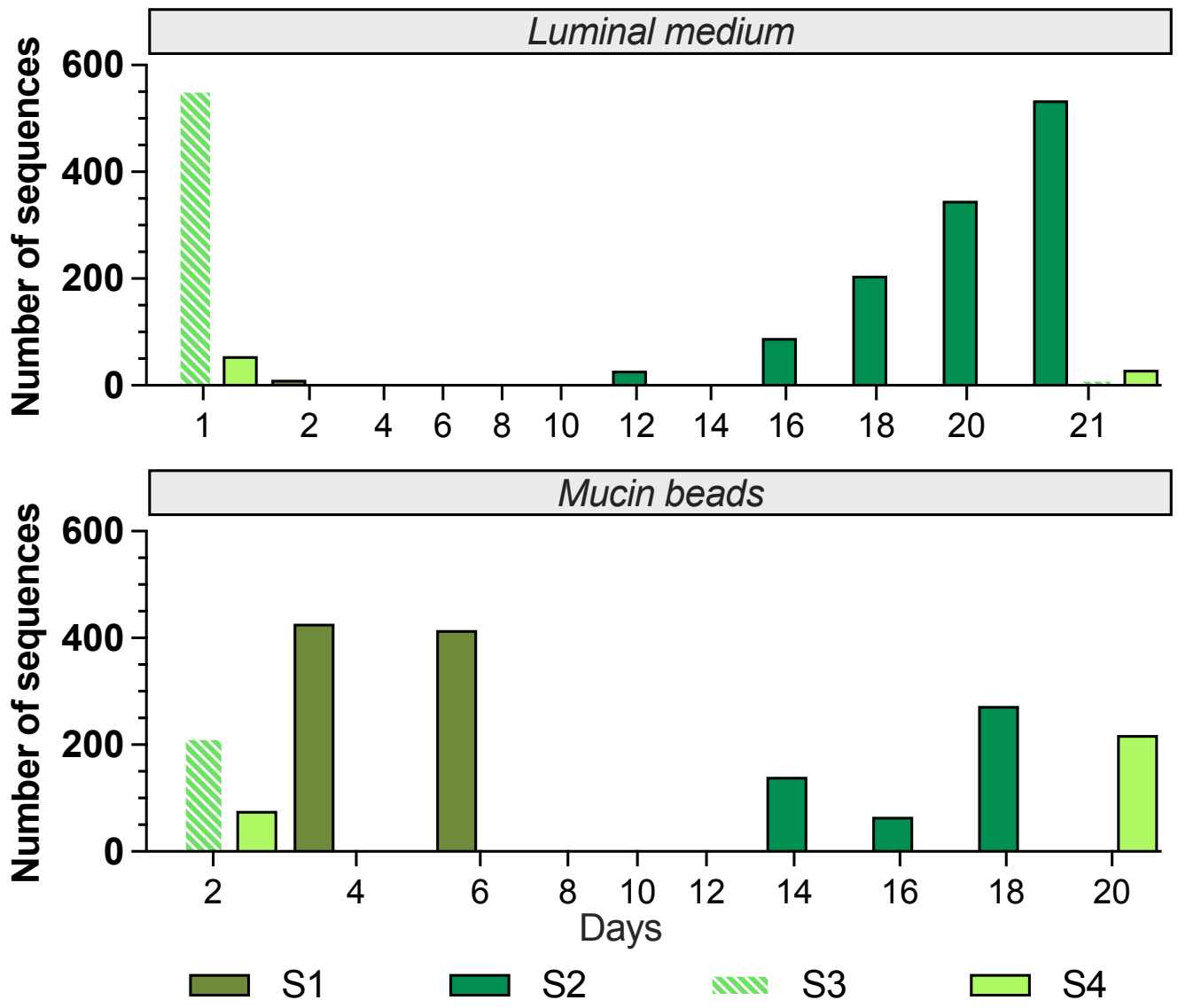

Supplemental Fig. S5 Detection of methanogenic *Archaea* in the CANIM-ARCOL

Fermentations were performed in the CANIM-ARCOL under three dog size conditions, when bioreactors were inoculated with fecal samples from 13 healthy dogs (n=4 small "S", n=5 medium "M" and n=6 large "L"). Luminal medium of bioreactors and mucin beads were sampled daily to monitor methanogenic *Archaea*. Results are expressed as archaeal sequence number (*Methanobrevibacter smithii* only) for small condition only, since no archaeal sequence was amplified from the medium and large samples.
